# Berbamine prevents SARS-CoV-2 entry and transmission

**DOI:** 10.1016/j.isci.2024.111347

**Published:** 2024-11-08

**Authors:** Srikanth Sadhu, Sandeep Goswami, Ritika Khatri, Bharat Lohiya, Virendra Singh, Rahul Yadav, Vinayaka Das, Manas Ranjan Tripathy, Prabhanjan Dwivedi, Mitul Srivastava, Shailendra Mani, Shailendra Asthana, Sweety Samal, Amit Awasthi

**Affiliations:** 1 Immuno-biology Lab, Infection and Immunology Centre, Translational Health Science and Technology Institute, NCR-Biotech Science Cluster, Faridabad, Haryana 121001, India; 2 Immunology-Core Lab, Translational Health Science and Technology Institute, NCR-Biotech Science Cluster, Faridabad, Haryana 121001, India; 3 Infection and Immunology Centre, Translational Health Science and Technology Institute, NCR-Biotech Science Cluster, Faridabad, Haryana 121001, India; 4 Small Animal Facility, Translational Health Science and Technology Institute, NCR-Biotech Science Cluster, Faridabad, Haryana 121001, India; 5 Non-communicable Disease Centre, Translational Health Science and Technology Institute, NCR-Biotech Science Cluster, Faridabad, Haryana 121001, India

**Keywords:** health sciences, virology, drugs

## Abstract

Effective antiviral drugs are essential to combat COVID-19 and future pandemics. Although many compounds show antiviral *in vitro* activity, only a few retain effectiveness *in vivo* against SARS-CoV-2. Here, we show that berbamine (Berb) is effective against SARS-CoV, MER-CoV, SARS-CoV-2 and its variants, including the XBB.1.16 variant. In hACE2.Tg mice, Berb suppresses SARS-CoV-2 replication through two distinct mechanisms: inhibiting spike-mediated viral entry and enhancing antiviral gene expression during infection. The administration of Berb, in combination with remdesivir (RDV), clofazimine (Clof) and fangchinoline (Fcn), nearly eliminated viral load and promoted recovery from acute SARS-CoV-2 infection and its variants. Co-housed mice in direct contact with either pre-treated or untreated infected mice exhibited negligible viral loads, reduced lung pathology, and decreased viral shedding, suggesting that Berb may effectively hinder virus transmission. This broad-spectrum activity positions Berb as a promising preventive or therapeutic option against betacoronaviruses.

## Highlights

• Berbamine (Berb) exhibits antiviral activity against betacoronaviruses
• Berb reduces viral load via spike mediated entry and enhancing the host antiviral response
• Combination of Berb with remdesivir, clofazimine, and Fcn nearly eliminates viral load
• Co-housing experiments suggest potentiality of Berb to reduce virus transmission

## Graphical abstract

**Figure:**
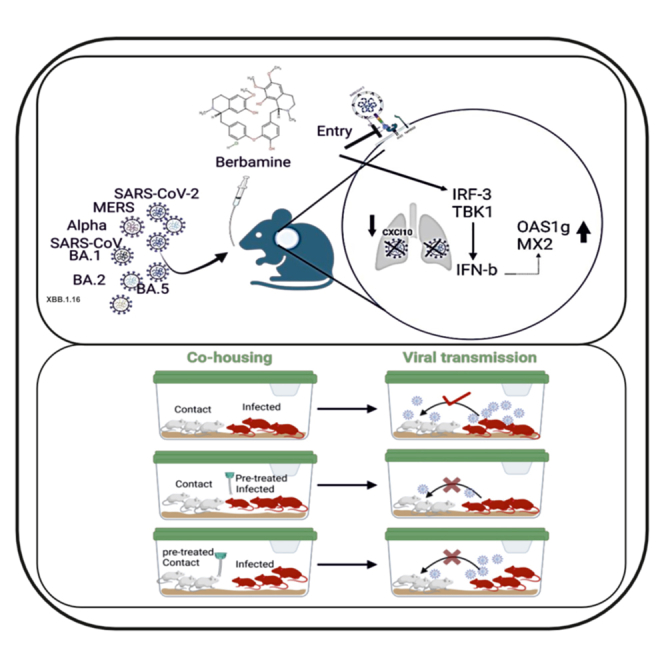

## Introduction

Severe acute respiratory syndrome coronavirus 2 (SARS-CoV-2) belongs to the *Coronaviridae* family, which includes both human and animal viruses, as well as SARS-CoV and MERS-CoV.^1^ Coronaviruses (CoVs) cause a range of illnesses from mild to severe disease, leading to major outbreaks such as SARS-CoV in 2002, MERS-CoV in 2012, and SARS-CoV-2 2019, each with varying mortality rates.^2^^,^^3^ The occurrence of three coronavirus pandemics—SARS-CoV, MERS-CoV, and SARS-CoV-2—happened at different intervals, raises concerns about the potential for future pandemics.

Throughout the SARS-CoV-2 pandemic, the rising number of COVID-19 cases posed an ongoing global health challenge, made even more difficult by the emergence of new variants. While the vaccination has been instrumental in controlling the pandemic, the development of antiviral drugs remains crucial, particularly for managing viral replication in immunocompromised individuals. Antivirals are also vital for addressing new variants such as, XBB.1.16, which continues to pose health risks by evading vaccine-induced immunity.^4^ The World Health Organization (WHO) designated XBB.1.16 as a variant under monitoring on March 30, 2023.^5^ Despite the progress made with vaccines and treatments the potential for viral mutations to diminish vaccine effectiveness underscores the needs for ongoing research into novel COVID-19 therapies and broad-spectrum antivirals for future pandemics.

To effectively manage COVID-19, potent antiviral agents are necessary, including both direct-acting antivirals (DAAs) and host-directed antivirals (HDAs).^6^ Remdesivir (RDV) and other antivirals, such as Paxlovid and Molnupiravir, have been approved for treatment of SARS-CoV-2.^7^^,^^8^^,^^9^^,^^10^ Research shows that SARS-CoV-2 exploits host pathways, primarily the ACE2 receptor, during its life cycle.^11^^,^^12^ The spike glycoprotein facilitates viral adhesion and entry, relying on host enzymes for cleavage to infect adjacent cells.^12^ The spike protein is essential for viral entry, causing membrane fusion and syncytia formation, which significantly impacts pathogenicity and transmission.^13^ There is growing interest in developing inhibitors that target both viral entry and replication. The strong binding of SARS-CoV-2 spike protein to the ACE2 receptor on host cells, which is crucial for viral replication, presents a valuable target for prevention.^12^ Entry inhibitors for SARS-CoV-2 are particularly promising due to their broad-spectrum effectiveness, complement existing treatments, and offering options for unvaccinated individuals. These inhibitors are especially important for immunocompromised patients, who are at higher risk for viral infections and require prophylactic and therapeutic options. Natural compounds with antiviral properties that boost immune responses and protect against co-morbidities are ideal candidates for these patients.

Plant-derived natural compounds have been instrumental in drug discovery due to their diverse targets, bioactivities, and low toxicity. Over 50% of approved drugs and candidates in the last 30 years are derived from natural compounds.^14^^,^^15^ Berb is a bis-benzylisoquinoline alkaloid from traditional Chinese medicinal plant *Berberis amurensis*, has shown potential as antiviral agent.^16^ *In silico* studies suggest that Berb inhibits SARS-CoV-2 by binding to spike protein and main protease, and disrupting ACE2-mediated endolysosomal trafficking via TRPMLs.^16^^,^^17^ However, these studies lack direct evidence of its effect on live SARS-CoV, MERS-CoV, SARS-CoV-2, and its variant including the newly emerging XBB.1.16. Additionally, *in vivo* studies are essential to validate its prophylactic and/or therapeutic efficacy in complex physiological environments. To address this, experiments were conducted to assess effectiveness of Berb against variants, such as Delta, Alpha, BA.1, BA.2, BA.5, and XBB.1.16, both *in vitro* and *in vivo*. Our results demonstrate the antiviral activity of Berb against SARS-CoV, MERS-CoV, SARS-CoV-2, Omicron variants, and XBB.1.16 in hACE2.Tg mice with a synergistic effect when combined with RDV, clofazimine (Clof.) and fangchinoline (Fcn). We also found that Berb prevents viral transmission *in vivo*.

## Results

### Berb effectively inhibits the entry of SARS-CoV-2 and its VOCs

**Figure 1:**
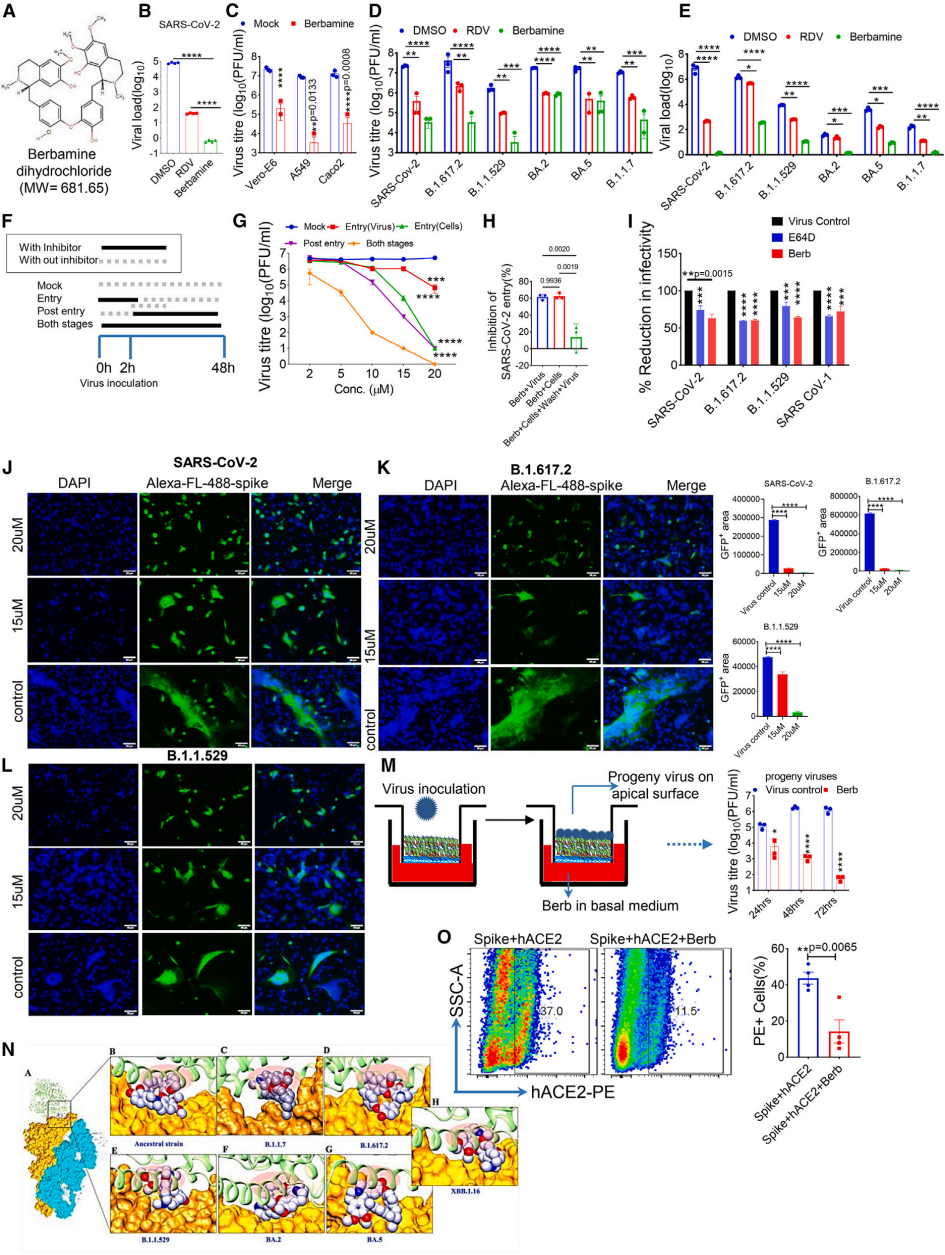
Berb inhibits SARS-CoV-2 and its VOCs entry (A) Chemical structure formula of Berbamine dihydrochloride and its molecular weight (MW). (B) Measurement of relative viral load between RDV and Berb by RT-PCR. ∗∗∗∗*p* < 0.0001 (One-way ANOVA; Tukey’s multiple comparison test). (C and D) Viral titer in VeroE6, A549 and Caco2 cells in the presence or absence of Berb (C), SARS-CoV-2 and its VOCs Supernatants were collected for quantification of viral titer by plaque assay. Data represent mean ± SEM, *n* = 3 biological replicates. two-way ANOVA followed by Tukey’s multiple comparison test (∗∗*p* < 0.01, ∗∗∗*p* < 0.001, ∗∗∗∗*p* < 0.0001); PFU, plaque-forming units (D). (E) Berb inhibited SARS-CoV-2 and its VOCs (multiplicity of infection (MOI) of 0.1) in veroE6 cells. Cell lysates were obtained to assess viral load, measured in relation to beta-actin. Data are mean ± SEM, *n* = 3 biological replicates. One-way ANOVA followed by Tukey’s multiple comparison test (∗*p* < 0.05, ∗∗*p* < 0.005, ∗∗∗*p* < 0.001, ∗∗∗∗*p* < 0.0001). (F and G) A time-of-addition assay was performed using Berb. VeroE6 cells were treated with Berb at different stages: during inoculation (entry stage), after inoculation (post-entry stage), or during both entry and post-entry stages of SARS-CoV-2 infection at an MOI of 3. The effectiveness of each treatment was assessed by measuring viral titers. The values shown are means ± SEM, of triplicate samples. ∗∗∗∗*p* < 0.0001 (two-way ANOVA followed by Tukey’s multiple comparison test). (H) Percentage entry inhibition of SARS-CoV-2. (I) VSV-based pseudotyped viral particle assay. Vero E6 cells were pre-treated with Berb and infected with SARS-CoV-2 S pseudotyped particles. Luciferase signals were quantified at 24 h after infection. Data are presented as mean ± SEM, *n* = 3 independent experiments. two-way ANOVA followed by Tukey’s-test (∗∗∗∗*p* < 0.0001). E−64D, a known coronavirus entry inhibitor, was used as a positive control. (J–L) Spike (SARS-CoV-2, B.1.617.2, and B.1.1.7) and hACE2 plasmids were co-transfected into BHK-21 cells, forming fusion structures observed through immunofluorescence. Data from three experiments in duplicates were analyzed for statistical significance using two-way ANOVA. Error bar represents mean values with ±SEM. Images were captured using an Olympus fluorescence microscope, and quantification was based on three randomly selected GFP+ areas. This experiment was repeated twice. Nuclei were stained with DAPI (blue), scale bar: 50 μm, magnification 20X. Statistical significance was determined via one-way ANOVA with multiple comparisons, using hACE2 expression as a control. Error bars depict mean values with ±SEM. ∗∗∗∗*p* < 0.0001. (M) Schematic representation of the SARS-CoV-2 infection experiment in an A549 airway tissue model. Cells were inoculated at the apical surface with SARS-CoV-2. Viral growth was monitored by titration of progeny virus in the mucus layer on the apical surface (two-way ANOVA followed by Tukey’s test; ∗*p* < 0.05, ∗∗∗∗*p* < 0.0001). (N) Molecular docking analysis of Berb on Ancestral, B.1.1.7, B.1.617.2, B.1.1.529, BA.2, BA.5 and XBB.1.16 variants. (A) Shows the binding of Berb at the interface of RBD-ACE2. (B–H) Insets show the close-up view of Berb binding at the interface. The stearic clash between Berb and superimposed structure of ACE2 is highlighted in transparent red, showing the hinderance caused by the inhibitor. The respective spike proteins of all strains are shown in Surf; Up conformation: orange and ACE protein is rendered in Lime; New cartoon, while Berb is shown in van der Waals (vDw) ice-blue, atom-type C: ice-blue and O:red. (O) Using flow cytometry, surface-expressed spike protein binding to soluble hACE2 was analyzed in HEK293T cells 36 h post-transfection (t test). The bar graph represents ±SEM. All experiments were performed twice to confirm the results. See also Figures S1 and S2 and Tables S1–S3.

To evaluate the antiviral potential of Berb (Figure 1A), we performed plaque-based infection assays using intestinal epithelial cells from hACE2.Tg mice infected with ancestral SARS-CoV-2. Berb treatment significantly reduced viral load compared to RDV (Figure 1B). Berb also effectively inhibited SARS-CoV-2 infection in Vero-E6, A549, and Caco2 cells (Figure 1C) without exhibiting cytotoxic effects at concentration ranging from 1.0 to 20 μM (Figure S1A, left and right panels). Further, a dose-dependent study of Berb against SARS-CoV-2 variants (B.1.617.2, B.1.1.529, BA.2, BA.5, and B.1.1.7) showed that, Berb rendered viral titers and viral loads undetectable (Figures S1B and S1C) with the logIC_50_ values reinforcing these results (Figures S2A–S2E). The WHO classified seven variants—Alpha, Beta, Gamma, Delta, Omicron, Lambda, and Mu—either variant of concern (VOCs) or variant of interest (VOIs) in 2022.^18^ Each showing amino acid changes in the spike protein that affects infectivity, transmissibility, pathogenicity, or antibody neutralization.^19^ Berb demonstrated broad antiviral activity, reducing viral titers and loads in both ancestral, and VOCs (B.1.617.2, B.1.1.529, BA.2, BA.5, and B.1.1.7) (Figures 1D and 1E).

SARS-CoV-2 virions are reported to be released into the culture supernatant between 8 and 12 h post-infection (hpi).^20^^,^^21^ A time-of-addition assay was conducted to examine the impact of Berb on the infection process, revealing its effectiveness at 15–20 μM (Figures 1F–1H). These findings suggest that Berb hinders SARS-CoV-2 infection by targeting both stages. To further explore how Berb blocks viral invasion, we used a vesicular stomatitis virus (VSV)-based spike glycoprotein (S) pseudo-typed virions-luciferase assay. The luciferase assay revealed that Berb significantly reduced the infectivity of pseudo-typed virions of SARS-CoV, SARS-CoV-2, and its VOCs (B.1.617.2, B.1.1.529), indicating its role in preventing SARS-CoV-2 entry (Figure 1I).

SARS-CoV-2 has shown to induce cell-to-cell fusion, enabling it to infect adjacent cells.^22^ We assessed syncytial formation in BHK-21 cells by co-expressing spike variants with ACE2.^23^ When spike variants (SARS-CoV-2, B.1.617.2, and B.1.1.529) were co-expressed with ACE2 in the presence of Berb, no syncytial formation (GFP+ area) was observed. In contrast, cells expressing Spike + ACE2 alone exhibited clear syncytial structures (GFP+ area), which was inhibited by Berb treatment (Figures 1J–1L). Since alveolar epithelia are essential for lung health and tissue repair, we used an *ex vivo* air-liquid interface (ALI) culture of A549 cells to mimic alveolar epithelia and test the effect of the Berb in infection model. A549 cells were infected with SARS-CoV-2 and treated with or without Berb via the basal medium (Figure 1M). Viral titers on the apical surface were significantly reduced at 24, 48, and 72 h in Berb treated samples, falling below the detection limit (Figure 1M right panel). These findings indicate that Berb inhibits viral entry though further investigation is required to elucidate precise mechanism.

To investigate the mechanism, we performed molecular docking studies to examine potential binding of Berb to the receptor-binding domain (RBD) of SARS-CoV-2 spike protein across various strains, including the ancestral strain and variants B.1.1.7, B.1.617.2, B.1.1.529, BA.2, BA.5, and XBB.1.16. Prior to docking, we compared interface site variations between RBD and ACE2 in each strain, noting significant differences between ancestral and Omicron strains. The docking studies revealed generally consistent binding affinities, with minor variations. Notably, the lowest docking score was observed for strain B.1.1.529 (−4.5), followed by the ancestral strain (−4.1), B.1.1.7 (−3.8), BA.5 (−3.9), XBB.1.16 (−3.8), B.1.617.2 (−3.2), and BA.2 (−3.1) (Table S1). Thermodynamic analysis using MM-GBSA to supported these results, showing the highest ΔG for the ancestral strain (−43.6 kcal/mol), while the other strains exhibited similar values (Table S2).

Structural analysis revealed that Berb occupied the RBD domain in all SARS-CoV-2 strains, blocking ACE2 (lime color) attachment through steric hindrance, suggesting a plausible mechanism of action (Figure 1N). Interaction analysis further identified key residues in the RBD, which Berb interacted across different strains (Figure 1N). These results suggest that Berb may have broad-spectrum activity against various SARS-CoV-2 strains (Table S3). To validate these results, we conducted an *in vitro* binding assay using spike glycoprotein-expressing HEK293T cells and soluble hACE2 protein either with or without Berb. Flow cytometry analysis revealed that the SARS-CoV-2 spike protein could potentially bind to soluble hACE2 (Figure 1O). However, spike-expressing cells treated with Berb demonstrated a ∼2– to 4-fold reduction in binding to soluble hACE2 (Figure 1O, right panel), indicating that Berb binds to the spike protein, thereby interfering with hACE2-mediated viral entry. Altogether we demonstrated that Berb exerts broad anti-SARS-CoV-2 activity by inhibiting spike-mediated viral entry and disrupting spike (S) and hACE2-mediated cell fusion activity.

### Berb exhibits antiviral synergy when combined with remdesivir, clofazimine and fangchinoline

Combinatorial therapy is essential for effectively controlling SARS-CoV-2 and emerging variants, as different drugs target various stages of infections. Given the potency of multiple antiviral drugs against SARS-CoV-2, we evaluated potential synergistic effects of Berb with known antivirals like RDV and Clof.^24^ Our study revealed that combining suboptimal doses of Berb (5 μM) and RDV (2.5 μM) significantly reduced viral titer of SARS-CoV-2 and its variants of concern (B.1.617.2, B.1.1.529, BA.2, and BA.5) (Figure S3A). We also examined synergy of Berb with Clof, which known to inhibit cell fusion and viral helicase activity. While suboptimal doses of either Berb (5 μM) or Clof (1 μM) alone did not reduce viral load, their combination resulted in a 3–4 log folds reduction compared to control (Figures S3B–S3D). Additionally, our recent study reported that Fcn is a potent pan-coronavirus inhibitor.^25^ To access the synergistic potential of Fcn with Berb, we treated the cells with suboptimal doses of both compounds (5 μM). The combinatorial treatment efficiently reduced the viral titer of SARS-CoV-2, including variants B.1.617.2, B.1.1.529, BA.1, and BA.5, whereas neither compound alone exhibited anti-viral activity (Figures S3E–S3G). The Bliss Index score was calculated to evaluate antiviral effect of these combinatorial treatments against SARS-CoV-2 and its variants (Table S4). Altogether, these findings suggest that Berb, in combination with RDV, Clof or Fcn, synergistically inhibits SARS-CoV-2 infection.

### Prophylactic administration of berb confers protection against SARS-CoV-2 infection in hACE2.Tg mice

**Figure 2:**
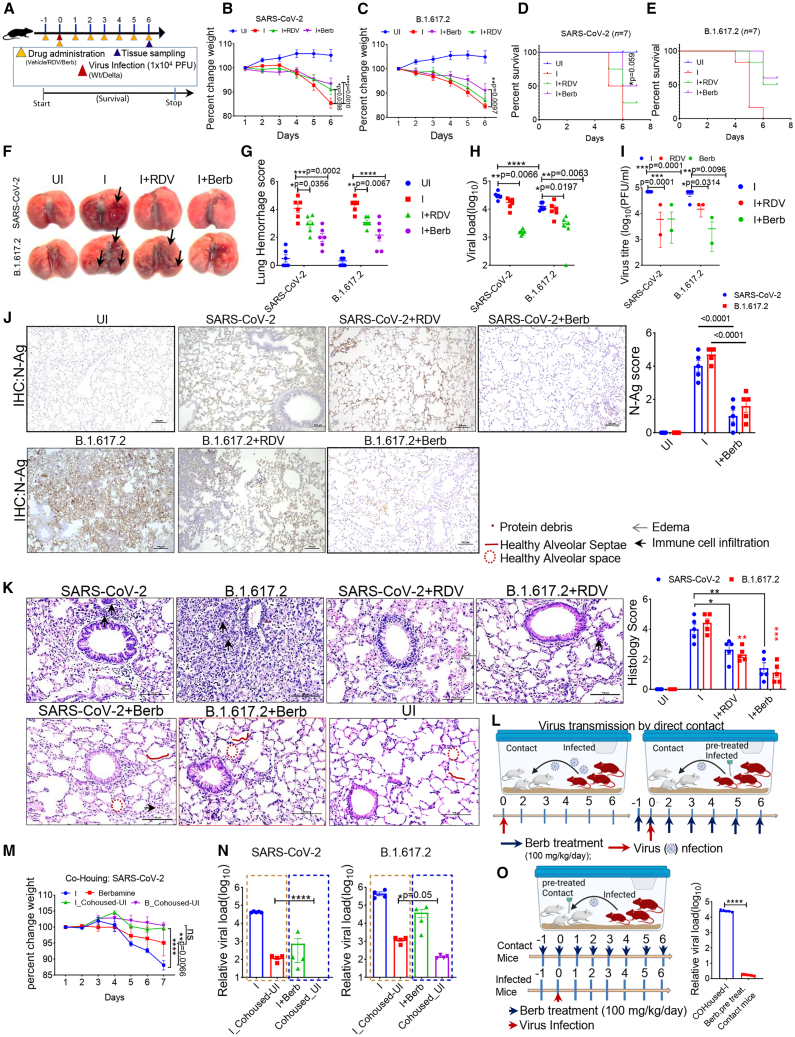
Prophylactic treatment of Berb controls viral burden and disease in SARS-CoV-2 infected mice (A) Experimental design for prophylactic treatment in hACE2.Tg mice: Mice received intranasal inoculation of SARS-CoV-2 and Delta variant (1 × 10^4^ PFU). Prophylactic treatment was performed 12 h before and immediately after infection (100 mg/kg; oral/day) till 6 dpi. RDV was used as a comparator drug. At 6 dpi, a group of mice (*n* = 6) was euthanized for tissue collection. Survival was monitored in another subset (*n* = 7). (B–E) Body weight change (two-way ANOVA followed by Tukey’s multiple comparison test) and percent survival (Mantel-Cox test) in uninfected (*n* = 7), SARS-CoV-2, and B.1.617.2-infected mice treated with Berb (100 mg/kg/day; oral), vehicle or RDV (25 mg/kg; i.p.) are shown (*n* = 7 for each group); ∗*p* = 0.059. (F) Image shows gross morphological changes in lungs. Black arrows represent hemolytic areas of the lungs. (G) Lung hemorrhage was scored on a 0–5 scale, where 0 represents a healthy pink lung, and 5 indicate a uniformly discolored dark red lung. The bar graph illustrates the mean ± SEM (*n* = 6 mice per group) and statistical analysis was conducted using one-way ANOVA followed by Tukey’s multiple comparison test; ∗∗∗∗*p* < 0.0001. (H and I) Viral load and viral titer was measured in control, vehicle, and Berb-treated mice. Error bar represents ±SEM (two-way ANOVA followed by tukey’s multiple comparison test). (J) Immunostaining for SARS-CoV-N antigen (brown) in lung tissue sections (*n* = 5 mice per group; experiment was performed once; 100 μm scale bar). Statistical significance was determined by two-way ANOVA followed by Tukey’s multiple comparison test. (K) Lung sections were subjected to hematoxylin and eosin staining, and the bar graph illustrates histology scores for various parameters, including hyaline membrane, septal wall thickness, septal degradation, neutrophil infiltration, and protein debris. The images were captured at 40× magnification, with a scale bar of 100 μm. The study included *n* = 5 mice per group, and statistical analysis was performed using one-way ANOVA followed by Tukey’s multiple comparison test. Bar graph represents ±SEM. (L–N) the experimental design is illustrated: four mice were infected with 1 × 10^4^ PFU of SARS-CoV-2 (infected mice), and another group (contact mice) was cohoused with them (L). Prophylactic Berb treatment was performed 12 h before and immediately after infection (100 mg/kg; oral/day). Each group comprised four infected and four contact mice. Measurements of percentage body weight change (M) and viral load (N) were measured at 6 dpi in infected mice and contact mice. Infected mouse groups received either vehicle or 100 mg/kg of Berb (oral). Values represent means ± SEM. Statistical analysis used two-way ANOVA followed by Tukey’s multiple comparison test, where ∗*p* < 0.05 and ∗∗∗∗*p* < 0.0001 indicate significance. (O) The experimental design is illustrated: four mice were infected with 1 × 10^4^ PFU of SARS-CoV-2 (infected mice), and another group (pre-treated contact mice; treated 12 h before cohousing till 6 dpi) was cohoused with them. Each group comprised four infected and four contact mice. Viral load was measured at 6 dpi in infected and contact mice. Contact mice groups received 100 mg/kg of Berb. ∗∗∗∗*p* < 0.0001; values represent means ± SEM. Statistical analysis used two-way ANOVA followed by Tukey’s multiple comparison test, where ∗∗∗∗*p* < 0.0001 indicates significance. All experiments were performed twice to confirm the results.

The antiviral activity of Berb was further evaluated in hACE2.Tg mice model for SARS-CoV-2.^26^ Mice were infected with SARS-CoV-2 and the Delta variant, a highly pathogenic strain,^27^^,^^28^ and then treated with Berb (100 mg/kg body weight, orally) 12 h before and immediately after infection^29^^,^^30^^,^^31^ (Figure 2A). Control mice received 1% DMSO as a vehicle. In both virus infections, we employed RDV (15 mg/kg body weight, intraperitoneally) was used as comparator, administered 12 h before and immediately after infection.^24^ At 6 days post infection (dpi), control mice lost over 20% of their body weight, while RDV-treated mice lost only 10% of their body weight in both the SARS-CoV-2 and Delta variant groups (Figures 2B and 2C). Mice treated with Berb in both infection groups lost only 5% of their body weight (Figures 2B and 2C). Consistent with these results, survival data also supported efficacy of Berb (Figures 2D and 2E). Furthermore, Berb treatment significantly reduced gross lung morphological changes, lung hemorrhage scores, viral load, and viral titer in the lungs were significantly reduced in both treatment groups compared to the RDV and vehicle-treated groups (Figures 2F–2I). Immunohistochemistry (IHC) data further supported these findings (Figure 2J). Histological analysis of lung tissues from infected hACE2.Tg mice showed interstitial pneumonia with diffuse lesions including thickened alveolar septa, alveolar space, edema, and infiltration of inflammatory cells into alveolar spaces (Figure 2K). In contrast, Berb-treated mice showed healthier alveolar spaces, alveolar septa, reduced immune cell infiltration, no epithelial cell degeneration and less pneumonia compared to both infected and RDV treated mice (Figure 2K).

Next, we assessed to test the effect of Berb on SARS-CoV-2 transmission using co-housing experiment as previously described.^32^^,^^33^ We infected hACE2.Tg mice with SARS-CoV-2 and divided them into two groups: group-1 received Berb treatment, and group-2 received a vehicle control. As noted earlier, Berb-treated mice exhibited better protection from SARS-CoV-2 infection, as evident by body weight measurements (Figure 2M). Both groups co-housed at 1:1 ratio with healthy hACE2.Tg mice (healthy contacts) in the same cage as indicated in Figure 2L. Healthy contact mice showed no significant weight difference between the two groups (Figure 2M). After 6-day co-housing period, we observed that healthy contact mice exposed to vehicle treated mice had a significant increase in viral load in their lungs, while those co-housed with Berb-treated infected mice had significantly lower lung viral loads (Figure 2N), indicating that Berb treatment effectively suppressed viral transmission. Similar results were obtained with Delta variant (Figure 2 N right panel), suggesting that Berb suppresses SARS-CoV-2 transmission across different strains. Additionally, in a separate experiment, healthy contact hACE2.Tg mice were treated with Berb for 6 days (12 h before and 24 h after co-housing), as indicated (Figure 2O), and Berb treatment again resulted in reduced viral transmission, as indicated by lower lung viral loads compared to the vehicle-treated control in healthy contact hACE2.Tg mice, as indicated by the lung viral load (Figure 2O, right panel). These findings collectively demonstrate that Berb effectively prevents SARS-CoV-2 transmission in hACE2.Tg mice.

### Berb retains antiviral activity against Omicron and its VOCs *in vivo*

**Figure 3:**
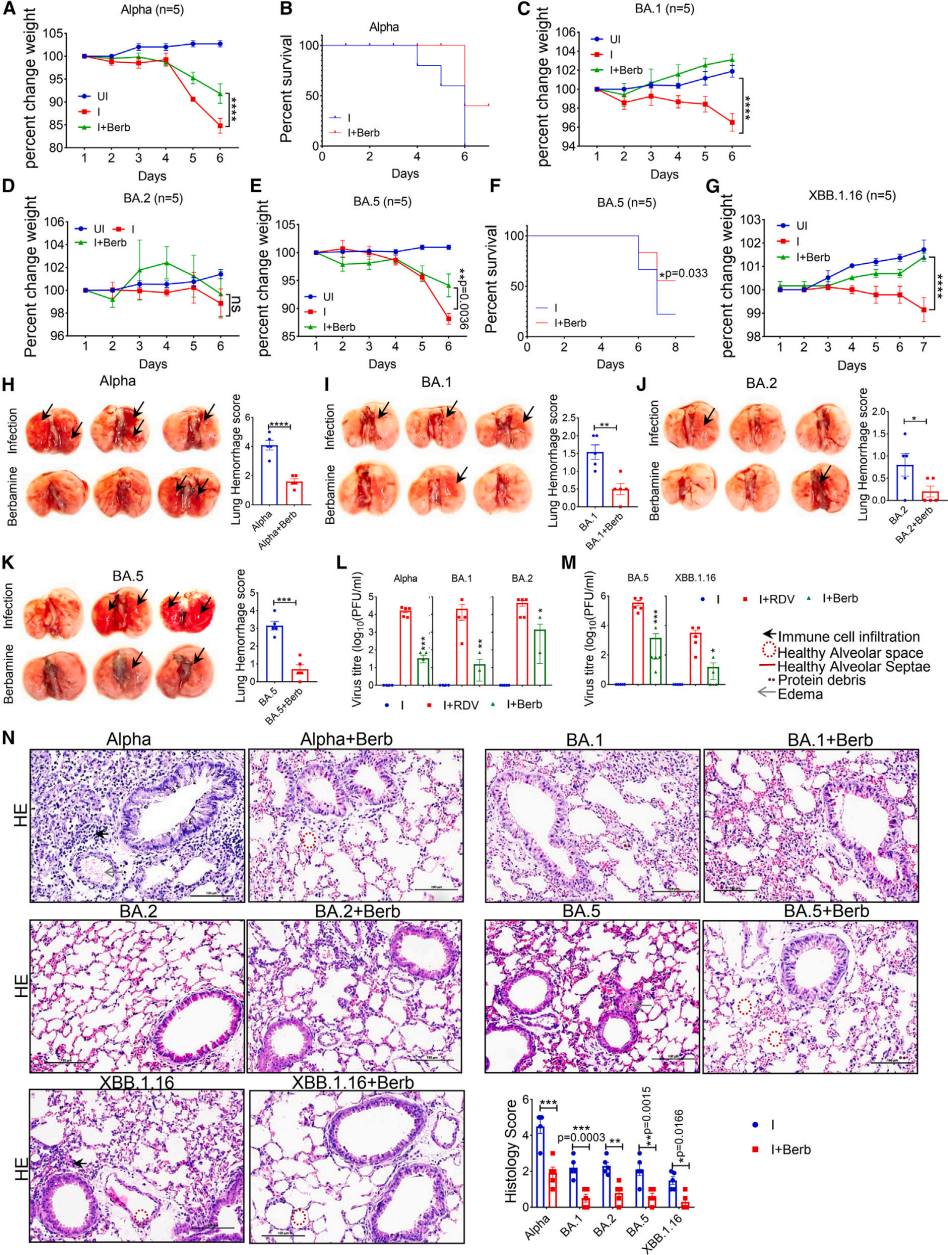
Berb retains antiviral activity against Alpha, BA.1, BA.2, BA.5, and XBB.1.16 *in vivo* Mice were intranasally inoculated with 1 × 10^4^ PFU of Alpha, and Omicron variants (BA.1, BA.2, and BA.5). The mice were treated with Berb (100 mg/kg) or vehicle (0 mg/kg) b.i.d. from the time of inoculation (0 hpi) to 5 dpi (*n* = 5 for each group). (A and B) Percentage change in weight (*n* = 5) and survival (*n* = 5) was measured in Alpha infected and Berb-treated mice (two-way ANOVA followed by Tukey’s multiple comparison test; ∗∗∗∗*p* < 0.0001). (C–F) Percentage change in weight (BA.1, BA.2, and BA.5) and survival (Wilcoxon test) was measured in BA.5 infected and Berb-treated mice. (G) Percentage change in weight was measured in XBB.1.16 infected and treated mice. The bar represents ±SEM. two-way ANOVA followed by Tukey’s multiple comparison test (body weight); ∗∗∗∗*p* < 0.0001. (H–K) The image shows gross lung morphological changes, and the bar graph represents lung hemorrhage score. 0–5, where 0 is a normal pink healthy lung, and 5 is a diffusely discolored dark red lung (black arrow indicates color of the lungs). Bar graph represents ±SEM. One-way ANOVA followed by Tukey’s multiple comparison test; ∗*p* < 0.05, ∗∗*p* < 0.005, ∗∗∗*p* < 0.0005, ∗∗∗∗*p* < 0.0001. (L and M) Virus titers in the lungs were measured by plaque assay. The values shown are means ± SEM. Statistical significance was measured by one-way ANOVA followed by Tukey’s multiple comparison test; ∗*p* < 0.05, ∗∗*p* < 0.005, ∗∗∗*p* < 0.0002. (N) Lung sections were subjected to hematoxylin and eosin staining, and the bar graph illustrates histology scores as mentioned previously. The images were captured at 40× magnification, with a scale bar of 100 μm. Two-way ANOVA followed by Tukey’s multiple comparison test. Bar graph represents ±SEM. See also Figure S3.

We also investigated the *in vivo* antiviral activity of Berb against various SARS-CoV-2 VOCs, including Alpha (lineage B.1.1.7) and Omicron (lineages BA.1, BA.2, BA.5 and XBB.1.5) in hACE2.Tg mice. Mice were remained susceptible to these VOCs, and the titers of Omicron in the lungs at 6 dpi being lower than those of other VOCs, consistent with previous reports.^34^^,^^35^^,^^36^^,^^37^^,^^38^^,^^39^ Notably, the Alpha strain was found to be ten times more lethal than the Omicron strain.^37^ Mice infected with the Alpha experienced severe decline body weight and survival rates, but Berb-treated mice did not show these effects (Figures 3A and 3B). Omicron BA.1, and BA.2 isolates were considerably less virulent,^40^ causing only 5% body weight loss in infected groups (Figures 3C and 3D). Interestingly, the BA.1-treated group showed slight body weight gain (Figure 3C), BA.2-treated mice did not (Figure 3D). The reduced pathogenicity of BA.1 and BA.2 aligns with previous reports.^34^^,^^41^ However, the BA.2-infected mice still had significantly lower body weight compared to uninfected controls (Figure 3C), whereas Berb-treated mice maintained their body weight (Figure 3D). Regarding the BA.5 variant, known for its high transmission efficiency and potential to cause severe illness,^36^ the percentage change in weight and mortality was significantly delayed compared with Alpha. Berb-treated mice showed reduced body weight loss and lower mortality (Figures 3E and 3F). BA.5-infected mice displayed more severe signs of illness (Disease score) than BA.1 and BA.2, but less than Alpha (Figures S4A–S4D).

The XBB.1.16 variant, classified by WHO official as a monitored variant in March 2023, did not cause significant weight loss in infected hACE2.Tg mice. Berb-treated mice infected with XBB.1.16 even showed weight gain comparable to uninfected controls (Figure 3G), and the disease index score was completely abolished in these mice (Figure S4E). Gross lung morphological changes, reduced lung lesions, and hemorrhage scores supported these findings (Figures 3H–3K).

Prophylactic administration of Berb decreased the viral load (Figure S4F) and viral titer (Figure 3L and 3M) across all examined VOCs. Mice treated with Berb also exhibited fewer lung histopathological lung signs compared to the infection groups (Figure 3N). These results suggest that Berb shows significant antiviral activity against Omicron and its VOCs, including XBB.1.16, *in vivo*.

### Therapeutic administration of berb control delta infection in mice

**Figure 4:**
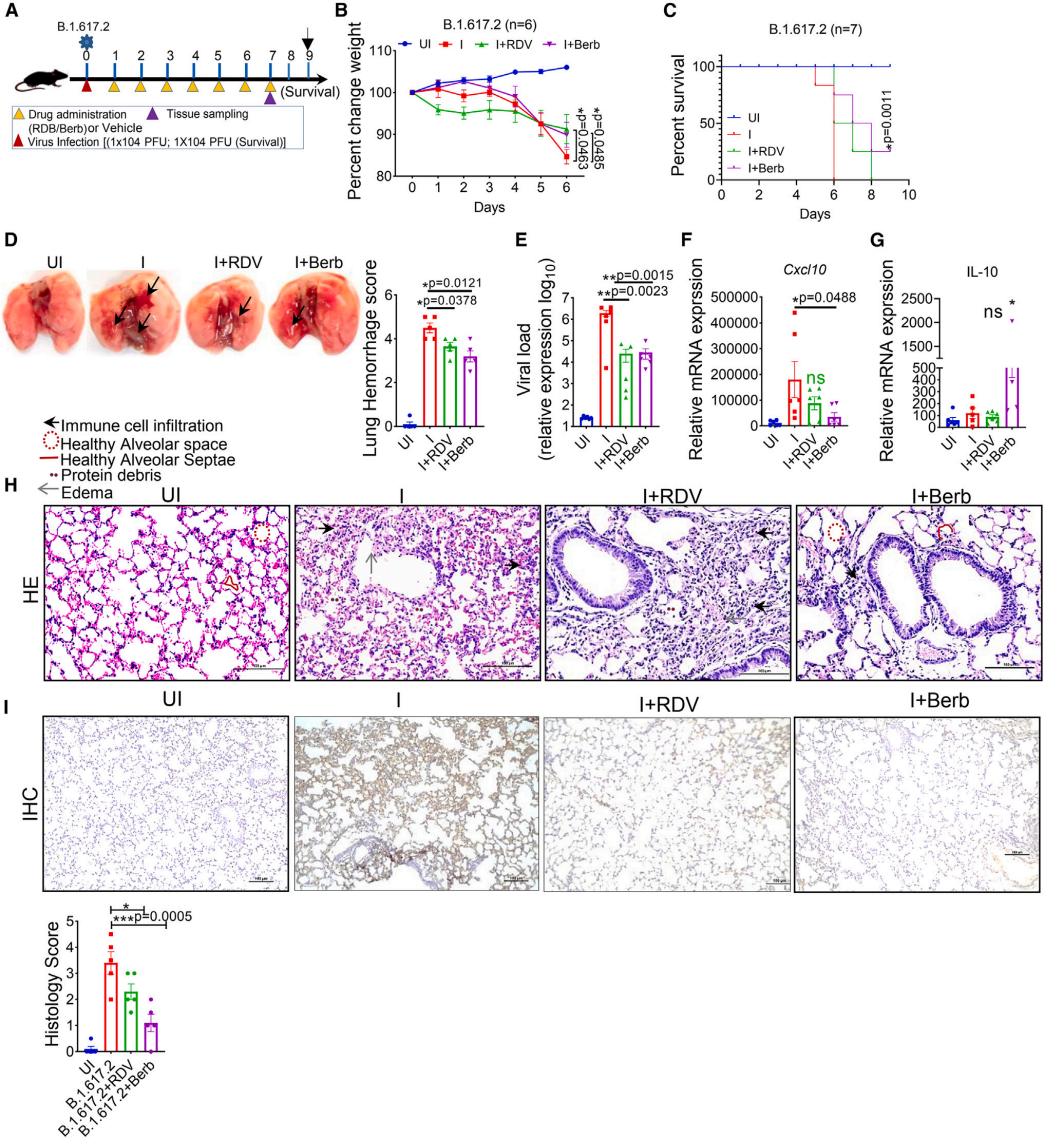
Therapeutic treatment with Berb decreased the viral load of SARS-CoV-2 and reduces disease severity in mice (A) The pictorial diagram shows the therapeutic treatment in a mouse model. Mice were intranasally inoculated with 10^4^ PFU of SARS-CoV-2 Delta variant. For therapeutic treatment, the mice were administration with Berb (100 mg/kg, oral; daily) or vehicle (1% DMSO in PBS) from 12 hpi to 6 dpi. RDV (15 mg/kg; i.p.) was used as a positive comparator drug. (B and C) Percentage change in body weight (*n* = 6 mice per group; two-way ANOVA; Tukey’s test) and percent survival (*n* = 7 mice per group; Wilcoxon test) were measured in delta variant infected and treated mice. (D) Image represents gross lung morphological changes in infected and Berb-treated mice (*n* = 5). The bar graph represents lung hemorrhage score. Values represents +SEM; One-way ANOVA followed by Tukey’s test. (E) Relative viral load was determined by RT-PCR and normalized to Actin-β. Bar graph represents +SEM (One-way ANOVA followed by Tukey’s multiple comparison test). (F and G) Relative mRNA expression analysis of CXCL10 and IL-10 was determined by RT-PCR; ∗*p* < 0.05. (H and I) Images represent H&E staining (H) and IHC (I) of the lung tissues, respectively. One-way ANOVA followed by Tukey’s multiple comparison test. Bar graph represents +SEM; *n* = 5 mice per group.

Next, to evaluate the therapeutic efficacy of Berb, we treated mice with Berb post infection to assess its effectiveness against B.1.617.2. Mice were treated with Berb (100 mg/kg body weight, orally), RDV (15 mg/kg body weight, intraperitoneally) or vehicle (1% DMSO in PBS) starting 12 h post infection (Figure 4A). By 6 dpi, the vehicle and RDV groups showed similar body weight losses of over 15% (Figure 4B). In contrast, Berb-treated mice experienced only 5–6% body weight loss and showed significant body weight recovery (Figure 4B). The survival rate in the Berb-treated group was superior to that of RDV-treated group (Figure 4C). Gross lung morphology changes and hemorrhage scores were consistent with the observed body weight and survival differences (Figure 4D). However, Berb and RDV administration had a limited effect on viral loads (Figure 4E).

Gene expression analysis indicated that Berb administration reduced the inflammatory response in hACE2.Tg infected mice (Figures 4F and 4G). These results suggest that, while delayed administration of Berb can decrease viral load and help in resisting infection, it is less effective than prophylactic treatment. Histopathological examination revealed that vehicle-treated mice exhibited immune cells infiltration, in the alveoli and alveolar walls (Figure 4H). In contrast such infiltration is markedly reduced in Berb-treated mice (Figure 4H). Immunological analysis showed extensive SARS-CoV-2 N-antigen staining in lungs of vehicle-treated mice (Figure 4I), whereas N-antigen staining was significantly reduced in Berb-treated animals (Figure 4I). Overall, these findings suggest that the therapeutic Berb administration effectively inhibits SARS-CoV-2 viral load and mitigates lung pathology associated with viral pneumonia, though it is not as effective as prophylactic treatment.

### Berb enhanced host antiviral response

**Figure 5:**
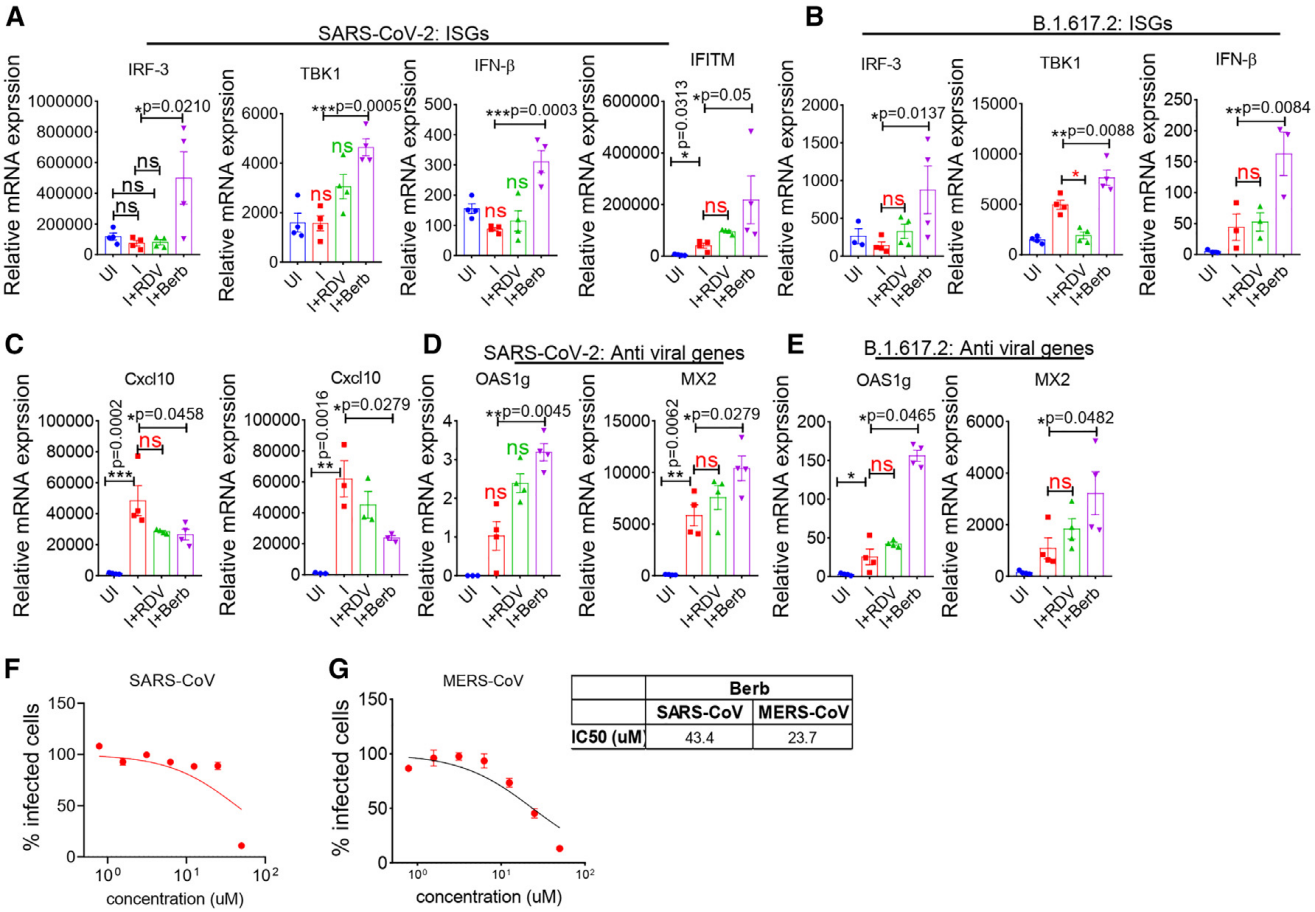
Berb promotes host mediated protective response RNA was isolated from infected (SARS-CoV-2 and B.1.617.2) and Berb-treated mice. cDNA was synthesized using applied biosystem c-DNA kit. (A and B) Relative mRNA expression of ISGs Irf3, Tbk1, IFN-β, and IFITM (one-way ANOVA; Tukey’s multiple comparison test). Bar graph represents ±SEM (ns = non-significant). (C) relative mRNA expression of chemokine CXCL10, measured by qPCR from lung samples (*n* = 4) (one-way ANOVA followed by Tukey’s, multiple comparison test); bar graph represents as a mean ± SEM. (D and E) antiviral genes (Oas1g and MX2) profile by RT-PCR from lung samples (*n* = 4) (one-way ANOVA followed by Tukey’s, multiple comparison test). (F and G) The effect of Berb on SARS-CoV and MESR-CoV virus inhibition was determined by neutralization assay. Table represents IC_50_ values. See also Figure S5.

Lastly, we evaluated host-directed immune response to infection in the presence of Berb treatment given our lung histopathological examinations showed substantial reduction in lung injury, immune cell infiltration, and overall histopathological score compared to SARS-CoV-2 and Delta strain infection (Figures 2 and 3). We focused on the key component of the host anti-viral immunity, including IFN-stimulated genes (ISGs), type I IFN and antiviral genes.^42^ Specifically, we examined the induction of IFNβ, ISGs such as interferon-induced transmembrane protein (IFITM), IFN regulatory factor 3 (IRF-3), IRF-7 and as well as antiviral genes, including myxovirus resistance 2 (MX2), MX1, 2′-5′ oligo adenylate synthetase 1 (OAS1g), OAS2 and OAS3 in context of SARS-CoV-2 and B.1.617.2 infections. The expression of ISGs typically follows prominent activation of IRF3 and IRF-7 during viral infections.^42^^,^^43^ To evaluate the effect of Berb treatment, we examined the expression of IRF3, IRF-7, and its downstream signaling molecule TANK-binding kinase 1 (TBK1). We observed that IRF-3 and TBK1 levels were significantly elevated in the Berb-treated group compared to the infection group (Figures 5A and 5B). Additionally, the relative expression levels of IFITM and IFN-β were markedly increased in the Berb-treated group compared to the infection group (Figures 5A and 5B).

We also assessed the chemokine CXCL10 (C-X-C motif chemokine ligand 10), which has been identified as robust biomarker for COVID-19 outcome with elevated levels linked to acute respiratory stress syndrome (ARDS) and neurological complications.^44^ CXCL10 levels were significantly reduced in Berb-treated group compared to the infection group (Figure 5C).

While OAS1g, MX2 levels were upregulated, MX1, OAS2, and OAS3 levels did not show significant changes (Figures 5D, 5E, S5A, and S5B). These findings suggest that IRF-3-TBK-1 pathway in Berb-treated mice stimulates IFN-β, IFITM, and antiviral genes (OAS1g, MX2) and downregulates CXCL10, thereby mitigating lung injury, viral pneumonia and inflammation compared to infection group. Furthermore, Berb inhibited SARS-CoV (IC_50_: 43.4) and MERS-CoV (IC_50_: 23.7) (Figures 5F and 5G), indicating its potential as an effective antiviral drug candidate against the *Coronaviridae* family. Overall, our data suggest that Berb is effective in combating SARS-CoV-2 infection, reducing lung injury and preventing pneumonia.

## Discussion

This study identifies Berb as a promising small-molecule inhibitor for SARS-CoV, MERS-CoV, and SARS-CoV-2, functioning both as direct acting antiviral (DAA) and through host-directed mechanisms. Our *in vitro* experiments demonstrated that Berb exhibits a potent antiviral activity against all VOCs, and *in vivo*, it is effective against the ancestral, B.1.617.2 (delta), and Omicron VOCs, including XBB.1.16. Berb inhibits syncytial formation and internalization of SARS-CoV-2, a critical step in viral—cell membrane fusion, and entry into target cells.^12^^,^^45^ The antiviral efficacy of Berb was confirmed in primary intestinal cells, VeroE6, Calu, Caco2 cells, as well as in A549 airway tissue model, without detectable toxicity. Furthermore, Berb treatment significantly reduced SARS-CoV-2-induced mortality, and provided robust protection against weight loss, lung pathology, and viral infection when administered either before the infection or 12 h post infection. These findings suggest that Berb could serve as an effective early treatment option against emerging SARS-CoV-2 VOCs.

Prophylactic Berb treatment significantly reduced lung viral load and improved body weight in mice, indicating its protective effect against SARS-CoV-2. Therapeutic treatment with Berb was insufficient for the complete lung recovery in SARS-CoV-2–infected hACE2.Tg mice, although they lost less body weight compared to vehicle controls. One possible reason for the weak therapeutic effect is that the drug might not effectively reach the site of viral proliferation in the mouse body. These results suggest Berb’s potential as a preventive option for high-risk individuals, demonstrating its antiviral effectiveness even in post-exposure therapy.

RDV, an approved antiviral, has shown mixed results in COVID-19 treatment, with limited clinical benefits in some cases and varied side effects.^7^^,^^46^^,^^47^ We show that therapeutic use of RDV as compared to Berb, in SARS-CoV-2 infection in hACE2.Tg mice, had minimal impact on reduction of viral load and body weight, suggesting that Berb has better antiviral activity.

Given the wide range of COVID-19 severity in humans, animal models (Hamster, hACE2.Tg transgenic mice and ferret model) that mimic the clinical features are essential for studying the disease pathogenesis, transmission, and evaluating treatments and vaccines.^48^ However, it is important to note that these models have limitations, and the results may or may not directly translate to human infections. Human populations exhibit various genetic backgrounds that influence susceptibility and immune response. SARS-CoV-2 infection is an acute infection and among all the models, the hACE2.Tg mice model is a reliable acute model for the SARS-CoV-2 infection.^49^ Virus transmission to cage-mates has also been observed in previous hamster and mice model studies.^32^^,^^50^ Previous studies have shown that naive hACE2.Tg mice co-housed with infected mice become seropositive by 14 dpi, and B.1.351-infected hACE2.Tg mice transmit replication-competent viruses to naive mice.^51^^,^^52^ Both hACE2.Tg mice and Syrian hamsters exhibit viral replication; however, severity of the SARS-CoV-2 infection varies significantly, with K18-hACE2 mice exhibiting more severe outcomes in comparison to Syrian hamsters.^53^^,^^54^ The hACE2.Tg mouse model is a considered a reliable acute model for SARS-CoV-2 infection causing lung inflammation and brain damage, which can be fatal.^49^^,^^55^. This acute fatality makes the hACE2.Tg model is preferable for antiviral studies, unlike hamsters and ferrets, which do not develop severe disease.^56^^,^^57^ We selected the hACE2 transgenic mouse model for its reliability, considering it well-suited for our study objectives. SARS-CoV-2 transmission has been demonstrated in both hamster and hACE2.Tg mouse model.^32^^,^^50^ Previous studies have reported that uninfected hACE2.Tg mice co-housed with SARS-CoV-2-infected mice can contract the virus.^51^^,^^53^ Consistent with previous findings, we found that Berb is able to suppress viral transmission to healthy contact hACE2.Tg mice.

Overall, our study presents that Berb is a promising SARS-CoV-2 inhibitor, effective against various strains, including VOCs, MERS-CoV, and SARS-CoV. Berb treatment significantly reduced lung injury, immune cell infiltration, and histopathological scores. Berb treatment upregulated type I IFN beta pathway to bolster host antiviral immunity. Notably, the Berb treatment reduced CXCL10 levels, which are associated with severe COVID-19 outcomes. Additionally, increased level of signaling molecules IRF-3 and TBK1 observed in the Berb-treated group indicate activation of the IRF-3-TBK-1 pathway, enhancing the expression of IFN-β, IFITM, and OAS1g, which are crucial in combating viral infections.^42^^,^^43^^,^^58^

Moreover, Berb and its analogs may have broader applications in combating other common respiratory viruses, including established coronaviruses and influenza viruses. We anticipate Berb could be particularly useful for unvaccinated individuals or those of high risk severe disease from SARS-CoV-2 VOCs and emerging variants. The synergistic effect of Berb in combination with other antivirals positions it as a promising candidate for clinical evaluation.

### Limitations of the study

One of key limitations of this study is that the effect of Berb is only tested in acute infection model of hACE2.Tg mice, which may not fully recapitulate the range of immune responses seen in human COVID-19 infections. SARS-CoV-2 induced immunopathology and fatality in hACE2.Tg mice could attributed to leaky human ACE2 expression in brain and other body tissues, possibly leading to exaggerated inflammatory responses.^55^^,^^59^ Therefore, testing Berb antiviral efficacy in other models could provide different and better outcomes. Moreover, the molecular mechanism by which Berb enhances ISGs expression in SARS-CoV-2 infection is unclear, necessitating further study. In summary, our study demonstrated the promising antiviral activity of Berb through *in vitro* and *in vivo* experiments, further studies in advanced preclinical models and clinical settings are required to validate its utility against SARS-CoV-2 in human populations.

## Resource availability

### Lead contact

Further information and requests for resources and reagents should be directed to and will be fulfilled by the lead contact Dr. Amit Awasthi (aawasthi@thesti.res.in).

### Materials availability

This study did not generate new unique reagents. All the cell lines used in this manuscript will be made available upon request.

### Data and code availability

• All data supporting the findings are available within the article and supplemental information.
• This study does not report any original code.
• Any additional information required to reanalyze the data reported in this paper is available from the lead contact upon request.

## Acknowledgments

Funding support was provided to the A.A. laboratory from THSTI intramural core grant and Translational Research Program (TRP). A.A. is recipient of GN Ramachandran National Bioscience Award. We acknowledge funding support from DBT-BIRAC for preclinical COVID-19 studies grant (BT/CS0100/06/22, BT/CS0054/05/21 and BT/CTH004/01/20). We acknowledge 10.13039/501100020622THSTI intramural funding support. We sincerely thank, Anna Mykytyn and Dr. Bart Haagman (Erasmus Medical Center, Rotterdam, Netherland) for MERS-CoV and SARS-CoV experiments. We acknowledge Experimental Animal Facility (EAF) and infectious disease research facility (IDRF) for their support in conducting experiments. We acknowledge Akash Goswami and Ayush Raj Tyagi for their help in ABSL-3 experimentation. SARS-CoV-2 and VoCs were obtained from BEI Resources, 10.13039/100000060NIAID.

## Author contributions

Conceptualization: S. Sadhu and A.A.; methodology: S. Sadhu, B.L., and R.K.; performed the experiments: S. Sadhu, S.G., R.K., B.L., V.S., R.Y., V.D., M.R.T., and P.D.; bioinformatics analysis: M.S. and S.A.; formal analysis and investigation: S. Sadhu., R.K., B.L., V.S., S. Samal and S.G; resources: A.A.; writing—original draft: S.Sadhu; writing—review and editing: A.A.; visualization: S. Sadhu; supervision of the study: A.A.; funding acquisition: S. Sadhu and A.A.

## Declaration of interests

All the authors read the manuscript and declare no conflict of interest.

## STAR★Methods

### Key resources table

**Table:** 

REAGENT or RESOURCE	SOURCE	IDENTIFIER
**Antibodies**		

anti-human ACE2 antibody	R&D Systems, USA	Cat#AF-933; RRID: AB_355722
PE-conjugated goat anti-human secondary antibody	Jackson Immuno Research, USA	Code: 109-117-008; RRID: AB_2632442

**Bacterial and virus strains**		

SARS-CoV-2	BEI	USA-WA1/2020
B.1.617.2	BEI	hCoV-19/USA/PHC658/2021
B.1.1.529	BEI	hCoV-19/USA/MD-HP20874/2021
Alpha	Gift from Dr. Sweety, THSTI	N/A
BA.2	Gift from Dr. Sweety, THSTI	N/A
BA.5	Gift from Dr. Sweety, THSTI	N/A
MERS-CoV	European Virus Archive Global	NC_019843
SARS-CoV	Gift from CV Bart L Haagman	N/A
XBB.1.16	Gift from Dr. Shailendra Mani	N/A

**Chemicals, peptides, and recombinant proteins**		

PMA	Sigma Aldrich	CAS Number 16561-29-8
RPMI 1640	Hi Media	Product code: AT150
DMEM	Hi Media	Cat#2023-24
FBS (Fetal Bovine Serum)	Hi Media	RM9955-500ML
Gentamycin	Gibco	Cat#15710-064
MTT	Sigma	Cat#475959
FuGENE	Promega	Cat#E2311
E−64D	Sigma	Cat#E8640
DAPI	Sigma	Cat#D9542
SS Agar	Hi Media	Cat#M108D
LB Media	Hi Media	Cat#M1245
L-Glutamine	Invitrogen	Cat#A2916801
Pen/Strep	Thermo Scientific	Cat#11360070
Tween 20	Sigma 9005-64-5	Cat#9005-64-5
Ionomycin	Sigma Aldrich	Cat# I9657
Golgi stop	BD	Cat# 555028
Berbamine Dihydrochloride	Sigma-Aldrich	CAS 6078-17-7.
TPCK-Trypsin	Thermo Fisher scientific	Cat# 20233
Remdesivir	MedChem	Cat# GS-5734

**Critical commercial assays**		

cDNA Synthesis kit	Applied Biosystem	Cat#43-688-14

**Experimental models: Cell lines**		

Caco2	Gift from CV Srikanth	N/A
VeroE6	ATCC	Cat#CRL-1586
HEK293T	RIKEN Bioresource	Cat#RCB2202
A549	Gift from Dr. Sweety	N/A
293T-hACE2	BEI Resources	NR52511
Primary Intestinal epithelial cells	Sadhu S et al., 2023	N/A

**Experimental models: Organisms/strains**		

K18-hACE2.Tg Mice 2B6.Cg-Tg(K18-ACE2)2Prlmn/J	The Jackson laboratory	Strain #:034860

**Oligonucleotides**		

N-Gene Forward and reverse primer	Sadhu S et al., 2023^32^	N/A
β-Actin Forward and reverse primer	Sadhu S et al., 2023^32^	N/A
Tbk-1 Forward and reverse primer	Sadhu S et al., 2023^32^	N/A
Irf-3 Forward and reverse primer	Sadhu S et al., 2023^32^	N/A
Ifitm Forward and reverse primer	Sadhu S et al., 2023^32^	N/A
Oas1g Forward and reverse primer	Sadhu S et al., 2023^32^	N/A
OAS2 Forward and reverse primer	Sadhu S et al., 2023^32^	N/A
Mx1 Forward and reverse primer	Sadhu S et al., 2023^32^	N/A
Mx2 Forward and reverse primer	Sadhu S et al., 2023^32^	N/A

**Recombinant DNA**		

pNL4-3. Luc. R-E−	Gift from Dr. Kalpana Luthra	N/A

**Software and algorithms**		

GraphPad prism v7	GraphPadSoftware, Sandiego, California USA	https://www.graphpad.com/scientific-software/prism/
MetaboAnalyst	Free online software	https://www.metaboanalyst.ca/
FlowJo Analyst	FlowJo Software (Treestar)	https://www.flowjo.com/
SDS 2.1 Software	Applied Biosystem 7500	https://www.thermofisher.com/in/en/home/life-science/pcr/real-time-pcr/real-time-pcr-instruments/7500-fast-real-time-pcr-system.html
FlowJo Analyst	FlowJo Software (Treestar)	https://www.flowjo.com/

### Experimental model and study participant details

This study was designed to characterize the antiviral activity of Berb using several variants of SARS-CoV-2. All SARS-CoV-2 experiments were conducted in a biosafety level 3 facility (IDRF; Infectious Disease Research Facility) at the Translational Health Science and Technology Institute. The virus infection in mice was performed in accordance with the Institutional Biosafety Committee and Institutional Animal Ethics Committee guidelines (THSTI/IAEC/262). Experiments were conducted at least twice, and the figure legends indicated all experiments with biological replicates.

#### Cell lines

Human lung epithelial cells (A549, kindly provided by Dr. Sweety), human small intestinal epithelial cells (Caco2, courtesy of Dr. CV Srikanth), 293T-hACE2 cells (BEI; NR52511), and human embryonic kidney cells (HEK293T; Cat#RCB2202) were cultured in Dulbecco’s Modified Eagle’s Medium (DMEM) containing 10% fetal bovine serum (FBS), 100 U/mL penicillin, and 100 μg/mL streptomycin. African green monkey kidney epithelial cells (VeroE6; ATCC Cat#CRL-1586) were maintained in Minimum Essential Medium (MEM) with 10% FBS, 2 mM L-glutamine, 100 U/mL penicillin, and 100 μg/mL streptomycin. All cell cultures were incubated at 37°C in a humidified atmosphere with 5% CO₂.

#### Viral strains

Betacoronavirus strains were utilized in this study. Infectious viruses were isolated through serial passaging in Huh7 and Vero E6 cells. All live-virus experiments were conducted within the high-containment ABSL3 and BSL-3 facilities at the Translational Health Science and Technology Institute (THSTI), following Institutional Biosafety and Animal Ethics Committee (IBSC and IAEC) guidelines (IAEC/THSTI/262). The virus strains used were as follows: SARS-CoV-2 (USA-WA1/2020) from BEI Resources, catalog no. USA-WA1/2020; SARS-CoV-2 B.1.617.2 (Delta variant) from BEI Resources, catalog no. hCoV-19/USA/PHC658/2021; SARS-CoV-2 B.1.1.529 (Omicron variant) from BEI Resources, catalog no. hCoV-19/USA/MD-HP20874/2021; SARS-CoV-2 Alpha, BA.2, and BA.5 Omicron subvariants, provided by Dr. Sweety at THSTI; MERS-CoV from the European Virus Archive Global, catalog no. NC_019843; SARS-CoV, received as a gift from Dr. CV Bart L. Haagman and SARS-CoV-2 XBB.1.16 variant, provided by Dr. Shailendra Mani. These strains were stored and handled following appropriate biosafety protocols.

#### Mice

All experiments were conducted at the Infectious Disease Research Facility (IDRF) in BSL-3 and ABSL-3 conditions, following Institutional Biosafety Committee (IBSC) guidelines. Virus challenge experiments were approved by the Institutional Animal Ethics Committee (IAEC; IAEC/THSTI/262), IBSC, and RCGM, in accordance with BRIC-THSTI and Government of India guidelines. Heterozygous K18-hACE2.Tg C57BL/6J mice (strain: 2B6.Cg-Tg(K18-ACE2)2Prlmn/J) were obtained from The Jackson Laboratory. The animals were housed and maintained at the THSTI Experimental Animal Facility (EAF) and fed a standard chow diet (Cat. no 1324p, Altromin; Germany) with water *ad libitum*. The mouse rooms were maintained at ∼19–26°C with ∼30–70% humidity and a 14-hour light/10-hour dark cycle. Mice were randomly allocated to experimental groups and housed individually. The number of animals used per experiment is specified in the figure legends. Throughout the investigation, mice were randomized to treatment groups, and littermates used in each experiment. Investigators were not blinded to group allocation and treatment in the cell and animal tests because the medication was administered to the mice and cells in distinct treatment conditions. Investigators ensured an unbiased approach to sex by including proportional representation of both male and female mice in each group.

### Method details

#### Primary intestinal epithelial cell line isolation and virus culture

Primary IECs were isolated from the Intestines of ACE2.Tg mice. Cold PBS with Gentamycin was used to clean the intestines. The intestines were split lengthwise, and the mucus layer was removed before immersing them in a 30 mM EDTA solution at 4°C for 20 minutes. PBS was used to gently remove colon epithelial cells. Following that, the cells were plated in collagen-coated plates.^32^^,^^60^

SARS-CoV-1 and MERS-CoV experiments were performed at Erasmus University, Rotterdam. We prepared infectious stocks by removing debris by centrifugation and passage through a 0.22-μm filter. The supernatant was then aliquoted and stored at −80°C at THSTI Infectious Disease Research Facility (Biosafety level 3 facility). The titers of the prepared virus stocks were determined as PFU per milliliter by a plaque assay.

#### Cytopathic effect (CPE) assay

In an Infectious Disease Research Facility (IDRF) in Faridabad, India, a SARS-CoV-2 CPE experiment was performed. Berb was titrated in DMSO before being sown in 96-well assay plates containing 10% DMEM medium. Further, Vero E6 epithelial cells were infected with SARS-CoV-2 (USA_WA1/2020) at a multiplicity of infection (MOI) of 0.01 In 2% FBS-containing medium. Cells were cultured for 48 h at 37°C, and the cytopathic affect was observed under the microscope.

#### MTT (3-[4,5-dimethylthiazol-2-yl]-2,5 diphenyl tetrazolium bromide) assay

VeroE6 Cells were seeded on a 96-well plate with 5000 cells/well for overnight cell adhesion. After 24 hrs, Berb was added from 0 to 20 μM and vehicle control: 1% Dimethyl sulfoxide (DMSO) as a control, and incubated for 24 hrs. Each dosage was repeated in triplicate. 10 μl of MTT (Sigma; #475989) (0.5 mg) solution was added, and the plate was placed back in the incubator for 3 hrs. Further, cell supernatant was collected 100 μl DMSO was added to each well, and the plate was placed on a shaker incubator for 1 hr. Absorbance was measured at 570 nm. Cell viability is calculated as the percentage change in treated cells' absorbance divided by untreated cells' absorbance.

#### Plaque assay

SARS-CoV-2, B.1.617.2, B.1.1.529, and its variants plaque assays were performed in Infectious Disease Research Facility (Biosafety level 3) at THSTI. VeroE6, Caco2 and A549 cells were infected with SARS-CoV-2, B.1.617.2, Omicron, and its variants at 0.1 MOI. After 1 hour of virus adsorption, the plates were washed once with DMEM without FBS, and the infected cells were treated with Berb (2, 5, 10, 15, 20 μM). Wells with 1% DMSO were used as virus controls. At 3 dpi with Omicron and 2 dpi with other variants, cells were fixed with 4% formaldehyde in phosphate-buffered saline (PBS) and stained with 1% crystal violet.

#### Immunofluorescence staining

BHK-21 cells were transfected with 0.5 μg of Spike- and/or ACE2-expressing plasmids in Opti-MEM medium using HD FuGENE transfection reagent (Promega, E2311). 2 hours post-transfection, inhibitors such as E-64d (20 μM, Sigma-Aldrich, E8640) and Berb (15, 20 μM) were added to the corresponding wells for the fusion inhibition assessment. The media was later replaced with complete DMEM. After 24 hours of transfection, cells were fixed in 4% paraformaldehyde and permeabilised in PBS with 0.1% Triton. Nonspecific binding was blocked for 1 hour at room temperature with 3% goat serum in PBS. The cells were then treated with anti-Spike polyclonal sera (1:200) overnight at 4°C. The next day, the cells were rinsed with PBS before being treated with Alexa Fluor 488-conjugated anti-rabbit immunoglobulin G (IgG) antibody (1:1000; Invitrogen, Thermo Fisher Scientific) as the secondary antibody. The cell nuclei were counterstained with DAPI (D9542, Sigma-Aldrich, USA) after washing. Fluorescence microscopy (IX-71, Olympus) was used to observe protein expression.^23^

#### Evaluation of antiviral efficacy of Berb in A549 airway tissue model

A549 cells were grown at an air-liquid interface after being seeded on collagen-precoated membranes of Millicell inserts. The cells were cultured for ten days to develop alveolar epithelial cell phenotypes and express cell type-specific markers. In two groups (one group had Berb added to the basal media, while the other did not), the apical regions of A549 cells were rinsed with culture media before being infected with 4000 PFU of SARS-CoV-2. After 40 minutes of incubation, the apical area was cleaned with incomplete DMEM (0% FBS) and the apical medium was replaced with fresh culture medium supplemented with 10% FBS. After 60 minutes, 200 μl of culture media were collected from the apical area for viral titration at 24, 48, and 72 hpi.^61^^,^^62^

#### Viral load determination by qRT-PCR

Tissue samples were weighed and homogenized of infected, treated, and uninfected controls. Homogenized (Trizol reagent; Invitrogen) samples were centrifuged at 3000 r.p.m for 5 min (4°C) and stored at -80°C till further use. RNA was extracted as per the manufacturer’s protocol.^32^ Relative copy number estimation of SARS-CoV-2 RNA has been described as following. RNA was reverse transcribed and amplified using the Bio-Rad cDNA reverse transcription kit. Amplification was accomplished over 42 cycles. Copies of SARS-CoV-2 nucleocapsid (N) gene RNA in samples were determined as follows. 200 ng of RNA was used as a template for reverse transcription-polymerase chain reaction (RT-PCR) to target a highly conserved region of the N gene (forward primer: ATGCTGCAATCGTGCTACAA; reverse primer: GACTGCCGCCTCTGCTC). Detection and sub-genomic RNA copy numbers were estimated by the Ct method.^32^ VeroE6 cells b-actin (F 5’-ATCGTGCGTGACATTAAGGAG -3’; R3’- AGGAAGGAAGGCTGGAAGAG-5’), Human b-actin (5’-CACCATTGGCAATGAGCGGTTC-3’; 3’- AGGTCTTTGCGGATGTCCACGT-5’) and mice-b-actin (F5’-CATTGCTGACAGGATGCAGAAGG-3’; 3’- TGCTGGAAGGTGGACAGTGAGG-5’) genes were used as an endogenous control for normalization through quantitative RT-PCR. The reaction mixture contained final concentrations of primers of 100 nM. To compute the relative gene expression, we used the previously applied formula (POWER (2,-CT)∗10000.^63^^,^^64^^,^^65^^,^^66^ The qPCR data was collected using Applied Biosystems 7500 RT-PCR equipment.

#### Entry and post entry inhibitor assay

VeroE6 cells were treated with Berb (0, 2, 5, 10, 15, 20 μM) during inoculation (entry stage), after inoculation (post-entry stage), or during both entry and post-entry stages of SARS-CoV-2 infection at an MOI of 0.5. The viral inoculum was removed, and the cells were washed three times with PBS. At 1 h after inoculation, the cells were incubated at 37°C in 5% CO_2_ for 48 hours. Cells were then fixed quantify the viral titer using a PRNT assay.

#### Pseudo virus entry assay

HEK293T cells were seeded in 6 well plates at a density of 0.4 x 10⁶ cells per well. After 24 hours, pseudoviruses were produced as follows. 293T cells were transiently transfected with Spike plasmid and backbone pNL4-3. Luc. R-E- in OptiMEM media using HD Promega FuGENE and grown in DMEM media at 37°C and 5% CO_2_. The supernatants were collected, centrifuged, aliquoted, and stored at -80°C after 48 hours of incubation. SARS-CoV-2, B.1.617.2, B.1.1.529, and SARS-CoV-1 were titrated in 293T-hACE2 cell lines. 293T overexpressed hACE2 cells (BEI Resources NR52511) were seeded for infection titer, and 100μl of pseudoviruses supernatant was employed for infection. Cells were lysed with Britelite Plus reporter substrate (Perkin Elmer) 48 hours after infection, and luminescence was measured using a PerkinElmer luminometer. For the virus entrance assay, 40,000 cells/well were seeded into 96-well plates, and after 4 hours, an equivalent viral supernatant with 75,000 relative luciferase units was chosen for infection.^23^^,^^67^

#### Protein structure retrieval and system preparation

The protein structure of ancestral strain PDB-ID 7KRR : resolution 3.50 Å Å,^68^ B.1.1.7; PDB-ID 7LWV : resolution 3.12 Å,^69^ B.1.617.2; PDB-ID 7W92 : resolution 3.10 Å,^70^ B.1.1.529: PDB-ID 7TB4 : resolution 3.29 Å,^71^ BA.2; PDB-ID 7Y1Y : resolution 3.30 Å,^72^ BA.5: PDB-ID 7Y21 : resolution 2.80 Å^72^ and XBB.1.16: PDB-ID 8IOU : resolution 3.18 Å^73^ were retrieved from the RCSB protein data bank. In each structure, the up-conformation was chosen for docking studies. Further, 6M0J (RBD bound with ACE2)^74^ structure was chosen to compare the docked structures. All structures were prepared using the Protein Preparation Wizard module of Maestro (Schrödinger Release 2022-1).^75^^,^^76^ In the preparation process, the hydrogen and bond orders were added using PRIME. The hydrogen bond (HB) optimization and restrained minimization was also carried out for both the systems using the OPLS3 force field model.^77^

#### Ligand retrieval and preparation

The molecule Berb was built using 2d-sketch module of Schrodinger.^78^ The ligands were prepared using LigPrep module of Schrodinger and optimization was performed using the OPLS3 force field. The force field was set the same as with the receptor in this process.^77^ The compounds were prepared at physiological pH conditions, desalted, tautomer’s were generated and finally the compounds were minimized.^79^

#### Molecular docking

The molecular docking was carried out using Glide module in Schrodinger. A grid was generated using the centroid of the residues present at the RBD domain of each spike protein which are critically involved in interactions with ACE2 protein. The docking was performed on the chain which has up conformation and the residues of each protein chosen for building grid is provided in Table S1. The docking was performed using the Glide XP (extra precision) method using OPLS4 force field. After the docking, the binding conformations were studied to elucidate the essential interactions between protein and ligand.

#### Prime MM-GBSA calculations

Post-docking minimizations have also been used to improve the geometry of the docking structures. The final poses from the Glide docking were post-processed by using the Prime MM-GBSA module. The results of the docking were then quantified on the consensus of docking scores and Prime MM-GBSA energy.^80^ Prime MM-GBSA uses a continuum solvation model for refinement. The solvation model VSGB2.1 (variable-dielectric generalized born model)^81^ is used here, which incorporates residue-dependent effects and water is the solvent. OPLS3e force field is applied and minimize sampling method is used to minimize all atoms in each residue. The MM-GBSA method is used to calculate the relative binding affinity of ligands to the receptor (in kcal/mol).^80^ Because the MM-GBSA binding energies are estimates of binding free energies (ΔGbind), a more negative value implies a better binding. Prime MM-GBSA calculates the energy of optimized free receptors, free ligand, and a complex of the ligand with a receptor. The formula is given below:

(Equation 1) ΔGbind = ΔGsolv + ΔEMM + ΔGSA

Where, ΔGsolv is the difference in GBSA solvation energy of the protein-ligand complex and the sum of the solvation energies for unbound protein and ligand. ΔEMM is the difference in the minimized energies between the protein-ligand complex and the sum of the energies of the unbound protein and ligand. ΔGSA is the difference in surface area energies of the complex and the sum of the surface area energies for the unbound protein and ligand.

#### Bliss Index score

To calculate the Bliss Index for assessing synergistic interactions between drugs, we followed the following steps: First, we determined the single-agent effects, which represent the response or effect of each drug when used alone. Next, we calculated the combination effects, which are the observed effects when the drugs are used together. We then calculated the expected additive effect using the Bliss Independence model, which assumes that the combined effect of drugs is the sum of their individual effects, adjusted for the probability of interaction. The formula used is:

Eadd=EA+EB−(EA·EB)

Where, EAE and EBE represent the effects of SO dosage of drug A and drug B, respectively. Further, we calculated the Bliss Index (BI), which compares the observed effect of the drug combination to the expected additive effect. The formula is:

BI=Eobs−Eadd (E_obs_: Observed; E_add_: Additive effect). Where a positive Bliss Index >0 indicates synergism, =0 indicates an independent effect, and a negative value <0 suggests antagonism.^82^

#### Mice infection, transmission and treatment

Animal studies were carried out following the institutional animal ethical guidelines (IAEC). Animals were housed in groups and fed standard chow diets. 8-10 weeks old mice were intranasally inoculated with 1x10^4^ PFU of SARS-CoV-2, B.1.617.2 or PBS as a mock control (inside ABSL3 facility) under anesthesia with ketamine hydrochloride and Xylazine inhalation. Berb was suspended in 1% (w/v) DMSO contained PBS. Prophylactic treatment was administered 12 hours before and immediately after infection (100 mg/kg; oral/day),^29^^,^^30^^,^^31^ a route chosen for its ease of administration and similarity to the intended route in our study. Considering both the human PK studies and antiviral research, we opted for a Berb dosage of 100 mg/kg/day via the oral route in our study. Therapeutic treatment (100 mg/kg; oral/day) was initiated 12 hours after virus inoculation. Vehicle control mice were administered 1% (w/v) DMSO. Mice were observed daily for clinical symptoms and weight loss until the day of sacrifice. On day 7 post-infection, mice from each group were sacrificed and further examined for immunological and histological analysis.^56^^,^^83^^,^^84^^,^^85^ For virus titration and gene expression assays, lungs were collected from a subset of mice (n = 7) at 7 dpi and homogenized in 1ml Trizol. A part of the homogenate was subjected to plaque assays for virus titration, while another part of the tissue sample was minced in Trizol for relative viral load quantification. Total RNA was extracted from the homogenate using Trizol and analyzed by qRT-PCR as mentioned above.

For virus transmission between animals, 4 mice per cage were inoculated with 1 x10^4^ PFU of SARS-CoV-2 and the Delta variant and cohoused with 4 naïve mice (contact mice) in the same cage. In one group, infected mice were treated daily with Berb 12h before infection and 12hpi (as mentioned above), while in another group, infected mice were not pre-treated with Berb. Each treatment group consisted of four infected mice and four contact mice in one cage. Lung tissues were sampled from infected mice and contact mice at 7 dpi. Viral loads were analyzed by virus titration and qRT-PCR as described above.

All the experimental protocols were approved by the IAEC (Institutional Animal Ethics Committee) at THSTI (IAEC/THSTI/262) and were performed according to the guidelines of standard operating procedures of the biosafety level 3 animal facilities.

#### qRT-PCR

RNA was isolated from cell lysates, and lung homogenates by using Trizol method.^32^ For measurement of antiviral, chemokines and interferon stimulating gene expression, total RNA was then reverse-transcribed to cDNA using the iScript cDNA synthesis kit (Bio-Rad). Real-time PCR was performed as mentioned below. Briefly, by using SYBR green dye Kapa sybr fast qPCR Master Mix (2X) Universal kit on standard 7500 Dx real-time PCR system (Applied Biosystems), and analyzed the results by using software SDS2.1. To calculate the cycling threshold (CT), subtracted the CT value of the endogenous control gene β-actin from the CT value of each gene of interest. β-actin gene was used as an endogenous control for normalization. We used the previously applied formula (POWER (2,-ΔCT)∗10000 to calculate the relative gene expression. The acquisition of qPCR was done on Applied Biosystems 7500 RT-PCR systems.^63^ Hamster primers as follows – Mice primers as follows: Tbk1: 5’- GACATGCCTCTCTCCTGTAGTC-3 3′’-GGTGAAGCACATCACTGGTCTC-5’ ; IRF-3: 5’- CGGAAAGAAGTGTTGCGGTTAGC-3’ 3’-CAGGCTGCTTTTGCCATTGGTG-5’ ; IFN-b: 5’-CTTGGATTCCTACAAAGAAGCAGC-3’ 3’-TCCTCCTTCTGGAACTGCTGCA-5’ ; IFITM: 5’-GGCTTCATAGCATTCGCCTACTC-3’ 3’-AGATGTTCAGGCACTTGGCGGT-5’ ; Cxcl10: 5’-ATCATCCCTGCGAGCCTATCCT-3’ 3’-GACCTTTTTTGGCTAAACGCTTTC-5’ ; OAS1g: 5’-AGGAAAGGTGCTTCCGAGGTAG-3’ 3’-GGACTGAGGAAGACAACCAGGT-5’ ; MX2: 5’-ACCAGAGTGCAAGTGAGGAGCT-3’ 3’-GTACTAGGGCAGTGATGTCCTG-5’ ; β-actin-5’GATGTATGAAGGCTTTGGTC-3’ 5’-TGTGCACTTTTATTGGTCTC-3’ ; For VeroE6 cells β-Actin: 5’-ATCGTGCGTGACATTAAGGAG -3’; 3’- AGGAAGGAAGGCTGGAAGAG-5’.

#### Histopath and immunohistochemistry (IHC)

Lung tissues were harvested at 7 dpi and immersed in 10% formalin. Paraffin-embedded tissue samples were sectioned and stained with Hematoxylin & Eosin (HE) by the Histopathology laboratory (THSTI; Faridabad). The non-blinded histopathological severity score (Acute Lung injury score) was determined based on immune cell infiltration, damage to alveolar septae, thickness, alveolar space, and edema. The proportion of alveolar inflammation and Pneumonia in a particular area of a pulmonary section collected from each animal in each group was calculated using the following scoring method: 0, no pathological change; 1, affected area ≤ 10%; 2, affected area 10-50%; and 3, affected area >50%. Three individuals were randomly given the lung Injury scoring system to quantify histological features of ALI. They scored randomly chosen diseased lung tissue fields for immune cell infiltration, damage, proteinaceous debris, and alveolar septal thickening. The lung Hemorrhage score was also assigned on a scale of 0-5 (0 is a normal pink healthy lung, and 5 is a diffusely discolored dark red lung). This system helps understand the severity of ALI and can aid in developing effective treatments.

For immunohistochemistry, 4 μm sections were used as described earlier.^32^ Before epitope unmasking, paraffin-embedded sections were dewaxed and rehydrated for 15' in room temperature (RT) with xylene and gradient alcohol, respectively. Slides were then blocked with normal goat serum for 30 minutes at RT. The samples were treated overnight at 4°C with a primary antibody (SARS-CoV-2-N antigen (5-25 μg/ml; R&D, #1035145). At the same concentration, species-matched gamma globulin was utilized as an isotype control. After washing in PBST, sections were treated with species-matched secondary antibodies for 60 min at RT. The stained sections for the SARS-CoV-2-N protein were then examined and photographed using an Eclipse Nikon microscope.

### Quantification and statistical analysis

#### Statistical analysis

Statistical significance was determined using GraphPad Prism 8 version 8.4.3 (Graphpad software) to analyze the data. IC50 values were determined by normalizing the dilution curve to a virus (100%) and cell control (0%) and fitting in Graphpad Prism. The tests used to determine statistical significance are indicated in the figure legends. No data were removed from the analyses. For graphical renderings, bio render software was employed. The results were presented as + standard error of the mean (SEM). Only *p* values of 0.05 or less were considered statistically significant (non-significant: *p* > 0.5). Statistical details of the experiments can be found in the respective figure legends.
