# Supplementary material for: Berbamine prevents SARS-CoV-2 entry and transmission
Source: iScience. 2024 Nov 8;27(12):111347. doi: 10.1016/j.isci.2024.111347 (PMC11618033; doi:10.1016/j.isci.2024.111347)
Supplement: Document S1. Figures S1–S5 and Tables S1–S4 [file mmc1.pdf]

iScience, Volume *27*

Supplemental information

Berbamine prevents SARS-CoV-2 entry and transmission

Srikanth Sadhu, Sandeep Goswami, Ritika Khatri, Bharat Lohiya, Virendra Singh, Rahul Yadav, Vinayaka Das, Manas Ranjan Tripathy, Prabhanjan Dwivedi, Mitul Srivastava, Shailendra Mani, Shailendra Asthana, Sweety Samal, and Amit Awasthi

Supplementary Information

Table S1: Variations and similarity of the residues identified at the interface ACE2-RBD in Ancestral, B.1.1.7, B.1.617.2, B.1.1.529, BA.2, BA.5 and XBB.1.16 variants, related to Figure 1.

Ancestral	B.1.617.2	B.1.1.7	B.1.1.529	BA.2	BA.5	XBB.1.16
K417	K417	K417	N417	N417	K417	K417
G446	G446	G446	S446	G446	G446	S446
Y449	Y449	Y449	Y449	Y449	Y449	Y449
Y453	Y453	Y453	Y453	Y453	Y453	Y453
L455	L455	L455	L455	L455	L455	L455
F456	F456	F456	F456	F456	F456	F456
F486	F486	F486	F486	F486	V486	S486
N487	N487	N487	N487	N487	N487	N487
Y489	Y489	Y489	Y489	Y489	Y489	Y489
Q493	Q493	Q493	Q493	R493	R493	Q493
S494	S494	S494	S494	S494	S494	S494
G496	G496	G496	S496	G496	G496	G496
Q498	Q498	Q498	R498	R498	Q498	Q498
T500	T500	T500	T500	T500	T500	T500
N501	N501	Y501	Y501	Y501	N501	N501
G502	G502	G502	G502	G502	G502	G502
Y505	Y505	Y505	H505	H505	Y505	Y505

Table S2: showing the docking score and MMGBSA values of Berbamine on Ancestral, B.1.1.7, B.1.617.2, B.1.1.529, BA.2, BA.5 and XBB.1.16 variants, related to Figure 1.

Strain	Docking score	MM-GBSA (kcal/mol)
Ancestral	-4.1	-43.6
B.1.1.7	-3.8	-39.3
B.1.617.2	-3.2	-37.4
B.1.1.529	-4.5	-38.2
BA.2	-3.1	-34.4
BA.5	-3.9	-34.7
XBB.1.16	-3.8	-33.5

Table S3: The interacting residues within 4 Å of Berbamine on different strains, related to Figure 1.

Strain	Interacting residues
Ancestral	**K417,** I418, Y449, **Y453,** L455, **F456,** Y473, Q493, S494, G496
B.1.1.7	L455, F456, **E484,** Y489, F490, Q493
B.1.617.2	Y449, R452, L455, F456, Y489, F490, L492, Q493, S494, Y495, G496, Y505
B.1.1.529	N417, Y453, L455, F456, Y489, R493, **S494**, Y495, S496
BA.2	**R403**, **Y449**, Q493, S494, Y495, G496, **R498**, Y501, H505
BA.5	R403, G416, N417, Y453, L455, F456, R457, K458, **Y489,** Y501
XBB.1.16	R403, **Y449**, Y453, L455, F456, Q493, S494, Y495, G496, **R498**, Y501, H505

Table S4: Bliss Index Score for the combinatorial treatment in-vitro, related to Figure S3.

**Supplementary Table-04: Bliss Index Score for the combinatorial treatment in-vitro**											
	**Strain Name**	**Average Values**				**E_{eff}(alone)**		**E_{add}**	**E_{obs}**	**Bliss Index(BI)[E_{obs}-E_{add}]**	**Effect**
A		**DMSO**	**Berb(SO)**	**RDV(SO)**	**Berb(SO)+RDV(SO)**	**Berb(SO)**	**RDV(SO)**				
**Berb+RDV**	**SARS-CoV-2**	1.73×10^{8}	6.91×10^{7}	4.8×10^{7}	2.6×10^{6}	0.601	0.72	0.889	0.985	0.0957	Synergistic
	**B.1.617.2**	4.4086×10^{8}	2.814×10^{8}	1.0887×10^{8}	9.38×10^{6}	0.362	0.75	0.843	0.979	0.1361	Synergistic
	**B.1.1.529**	1.118×10^{7}	4.68×10^{6}	4.33×10^{6}	2.6×10^{5}	0.582	0.61	0.838	0.977	0.1386	Synergistic
	**BA.2**	17,90,933.33	5,39,733.33	662400	15,946.67	0.699	0.63	0.888	0.991	0.1027	Synergistic
	**BA.5**	2.69E+08	1.49E+08	4.72E+07	2169333.3	0.445	0.82	0.901	0.994	0.093	Synergistic
**B**		**DMSO**	**Berb(SO)**	**Clof(SO)**	**Berb(SO)+Clof(SO)**	**Berb(SO)**	**Clof(SO)**				
**Berb+Clof.**	**SARS-CoV-2**	1.74E+08	7.67E+07	7.47E+07	2133333.3	0.559	0.57	0.81	0.987	0.177	Synergistic
	**B.1.617.2**	3.473×10^{8}	2.1731×10^{8}	2.1418×10^{8}	4690000	0.374	0.38	0.614	0.986	0.372	Synergistic
	**B.1.1.529**	2.06267×10^{7}	4680000	3640000	346666.6	0.774	0.82	0.899	0.983	0.023	Synergistic
	**BA.2**	20,85,333	8,95,466	4,90,400	36,533	0.57	0.77	0.96	0.99	0.039	Synergistic
	**BA.5**	1.49213×10^{8}	4.6900×10^{7}	1.468×10^{7}	4.693×10^{6}	0.685	0.9	0.97	0.99	0.2	Synergistic
**C**		**DMSO**	**Berb(SO)**	**Fcn(SO)**	**Berb(SO)+Fcn(SO)**	**Berb(SO)**	**Fcn(SO)**				
**Berb+Fcn**	**SARS-CoV-2**	1.73867×10^{8}	8.6667×10^{7}	5.147×10^{7}	21,33,333	0.502	0.7	0.852	0.988	0.1357	Synergistic
	**B.1.617.2**	5.0652×10^{11}	2.3773×10^{11}	1.3924×10^{11}	2.7202×10^{10}	0.531	0.72	0.871	0.946	0.0756	Synergistic
	**B.1.1.529**	1864533.33	746933.33	797333.33	147200	0.599	0.57	0.829	0.922	0.0925	Synergistic
	**BA.2**	1.864	72392	897333	372500	0.6	0.57	0.82	0.98	0.151	Syner

		5×10^{7}	33	3				9			gistic
	**BA.5**	2.691 9×10^{8}	1.492 1×10^{8}	5.8273×10^{7}	3.5733 $\times10^{6}$	0.445	0.78	0.87 9	0.98 7	0.1084	Syner gistic
E_{add}: Additive effect; E_{obs}: Observed effect; Bliss Score=E_{obs}-E_{add} (>0 : Synergism; =0: Independent; <0: Antagonism)											

Supplementary Fig. 1

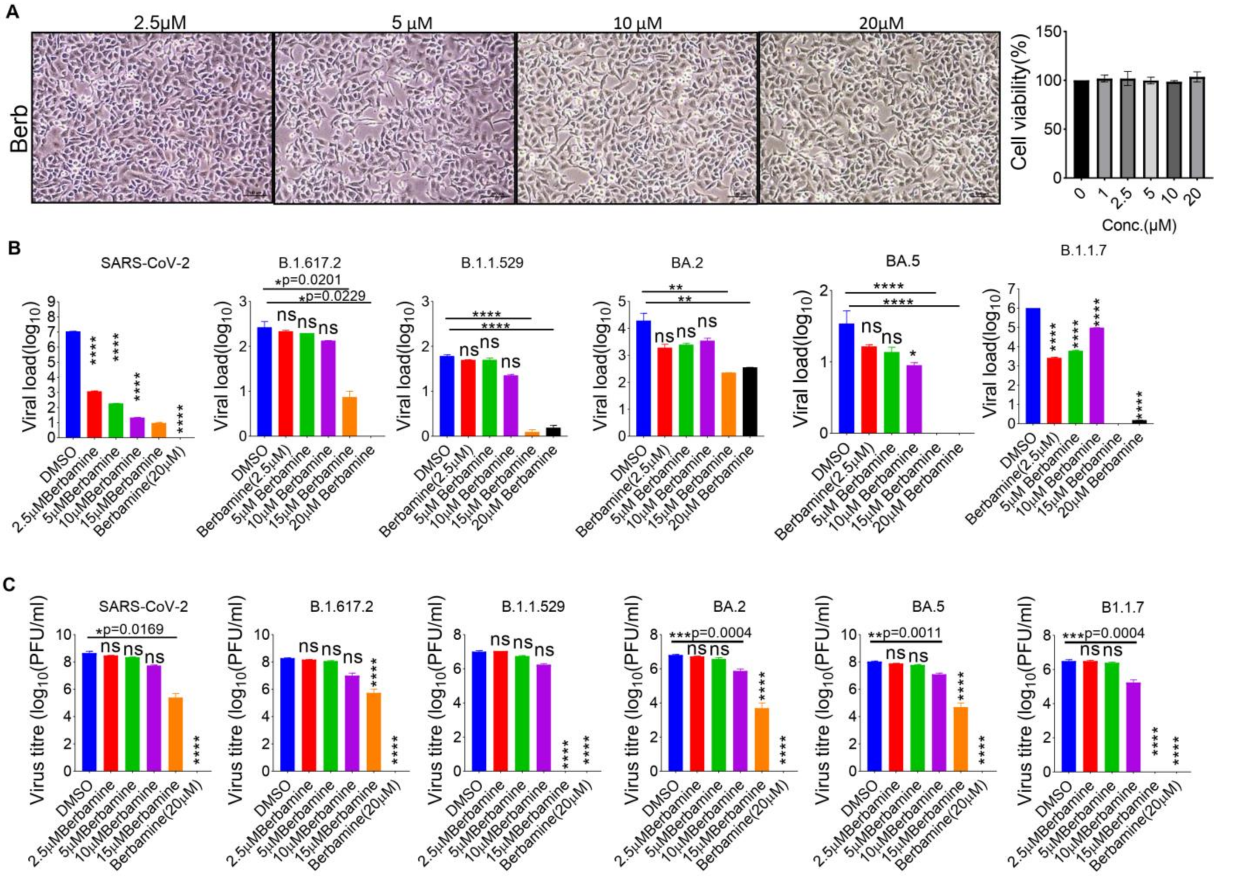

Figure S1. Berb inhibits SARS-CoV-2 and its variations in a dose-dependent manner, related to Figure 1. A) Images represent cytopathic effect of Berb on VeroE6 cells. Bar graph (right panel) represents the cell viability in the presence of Berb at different concentrations. Data represents + SEM; One-way ANOVA followed by Tukey's multiple comparison test (ns=non-significant). **B-C)** Viral load and viral titer was determined in the presence of Berb at different doses. Bar graph represents + SEM; One-way ANOVA followed by Tukey's multiple comparison test. All experiments were performed twice. Significant p values $p < 0.05$.

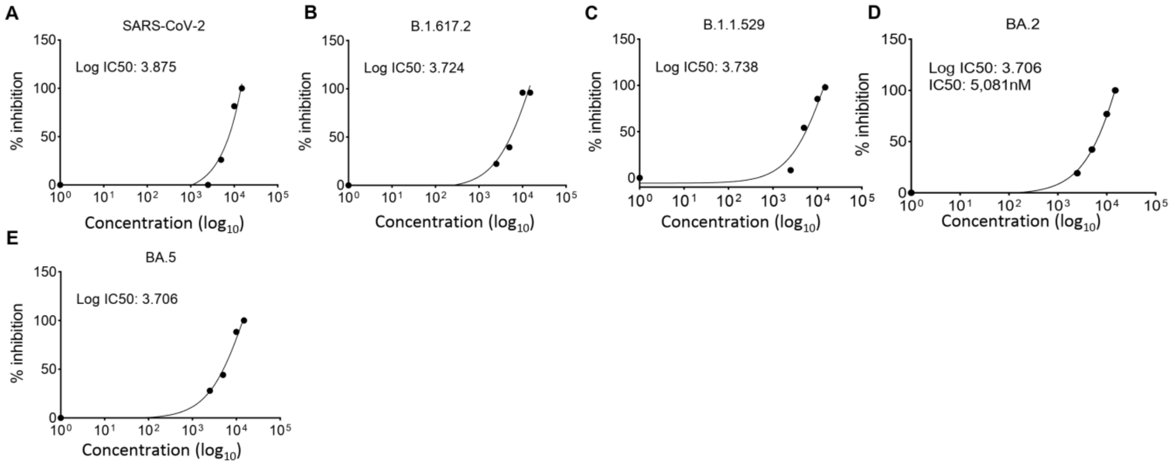

Figure S2. Berb IC50 values against SARS-CoV-2 and its VOCs, related to Figure 1. A-E) VeroE6 cells were infected with SARS-CoV-2 (0.1 MOI) and treated with Berb with different doses from 0 μM to 20 μM and further calculated the IC50 values based on the formation of the plaques.

Supplementary Fig. 3

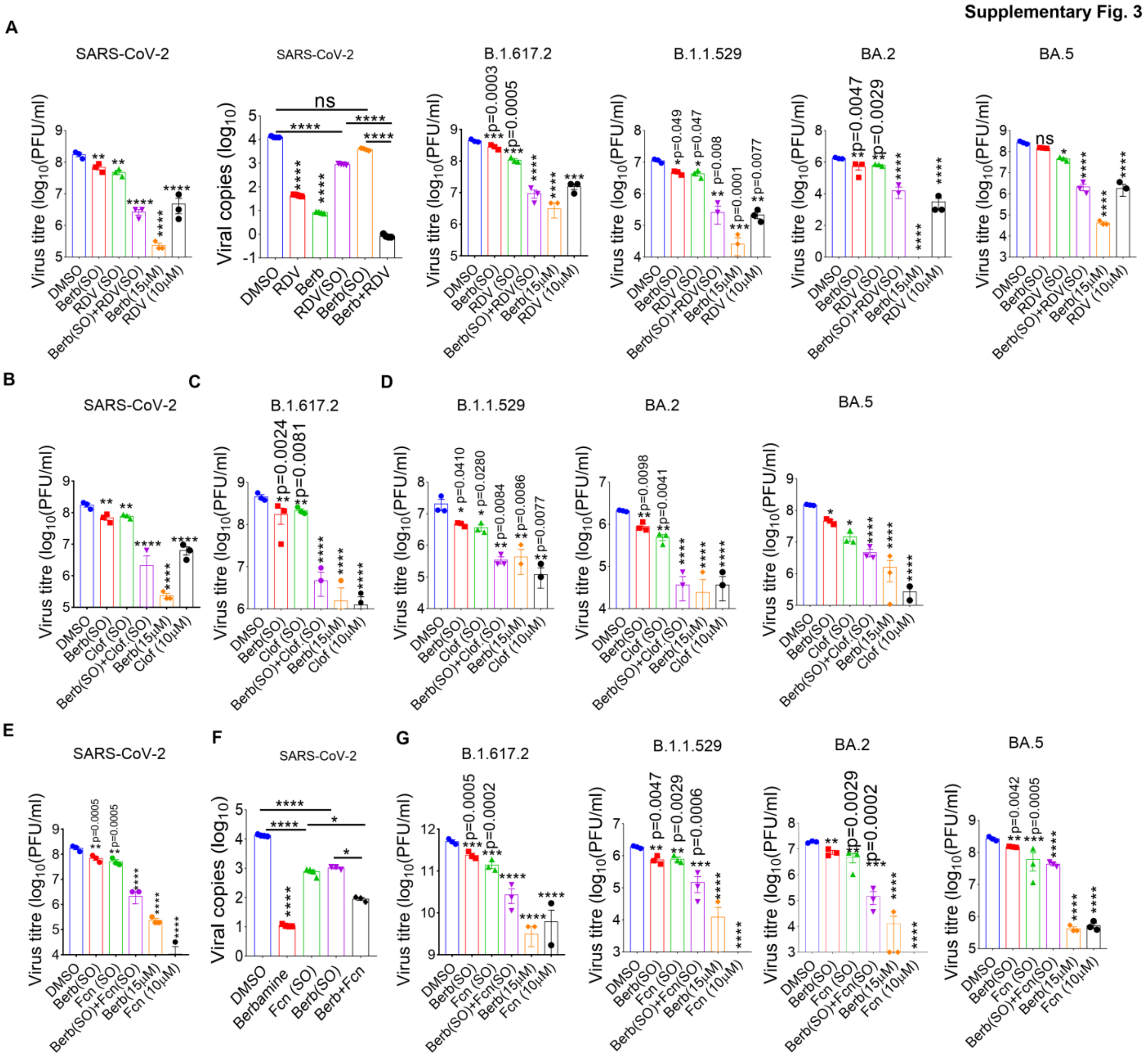

Figure S3. Berb exhibits antiviral synergy with Remdesivir, Clofazimine and Fangchinoline, related to Figure S3. A) To determine the synergistic effect of Berb with RDV, we treated infected veroE6 cells (SARS-CoV-2, B.1.617.2, B.1.1.529, BA.2, and BA.5) with the SO dose of Berb and RDV (5 µM + 2.5 µM). Relative viral load and Viral titer were determined by RT-PCR and plaque assay. Data represents ± SEM, n = 3; One-way ANOVA (Tukey's multiple comparison test). *p < 0.05, **p < 0.005, ***p < 0.0005, ****p < 0.0001. **B-C)** To determine the synergistic effect of Berb with Clof, we treated the infected veroE6 cells with SO dose of Berb and Clof (5 µM + 1 µM). Viral titer and relative viral load were determined by plaque assay and RT-PCR. Data represents ± SEM, n = 3. One-way ANOVA (Tukey's multiple comparison test); *p < 0.05, **p < 0.005, ***p < 0.0005, ****p < 0.0001. **D)** To determine the synergistic effect of Berb with Clof against B.1.617.2, BA.1 and BA.5, viral titer was determined by plaque assay. Data represents ± SEM, n = 3. One-way ANOVA followed by Tukey's multiple comparison test; ****P < 0.0001, ***P < 0.001, **P < 0.01, *P < 0.05 (for BA.5). **E-G)** To assess Berb synergy with Fcn, we treated SARS-CoV-2, B.1.617.2, B.1.1.529, BA.2, and BA.5 infected veroE6 cells with SO doses of Berb and Fcn (5 µM + 5 µM) or with these agents individually. Viral load was measured by RT-PCR and viral titer was measured by plaque assay. Data represents ± SEM, n = 3; One-way ANOVA (Tukey's multiple comparison test). All experiments were performed twice. Statistical analyses were compared with the DMSO-treated group; significant p values < 0.05.

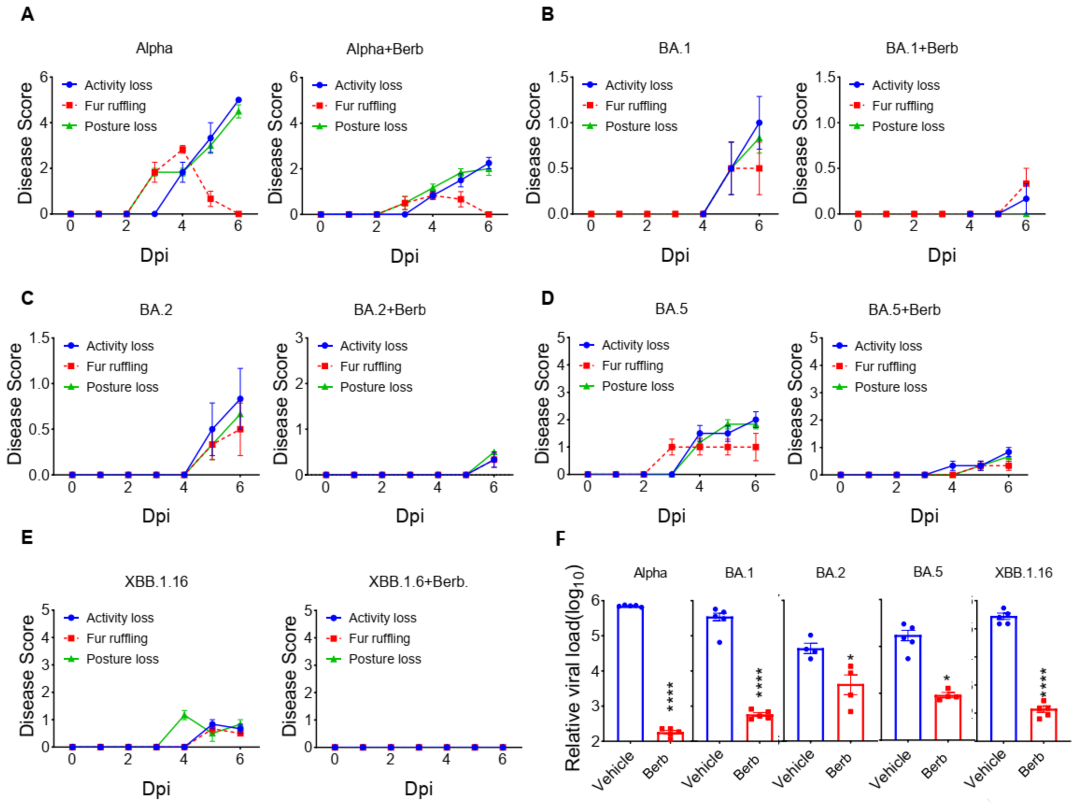

Fig. S4. Disease index score and viral load of mice treated with Berb in the context of Alpha, BA.1, BA.2, BA.5 and XBB.1.16, related to Figure 3. A-E) Mice were infected with Alpha, BA.1, BA.2, BA.5 and XBB.1.16. Every day, mice were observed for phenotypic characterization based on activity loss (0-5), fur ruffling (0-5), and Posture loss (0-5). Bar graph represents ± SEM; (n=5 mice per group). **F)** Relative expression of viral load was measured by RT-PCR. Data was normalized to Act-b. The values shown are means ± SEM. *P < 0.0445, ****p < 0.0001; paired t-test.

Supplementary Fig. 5

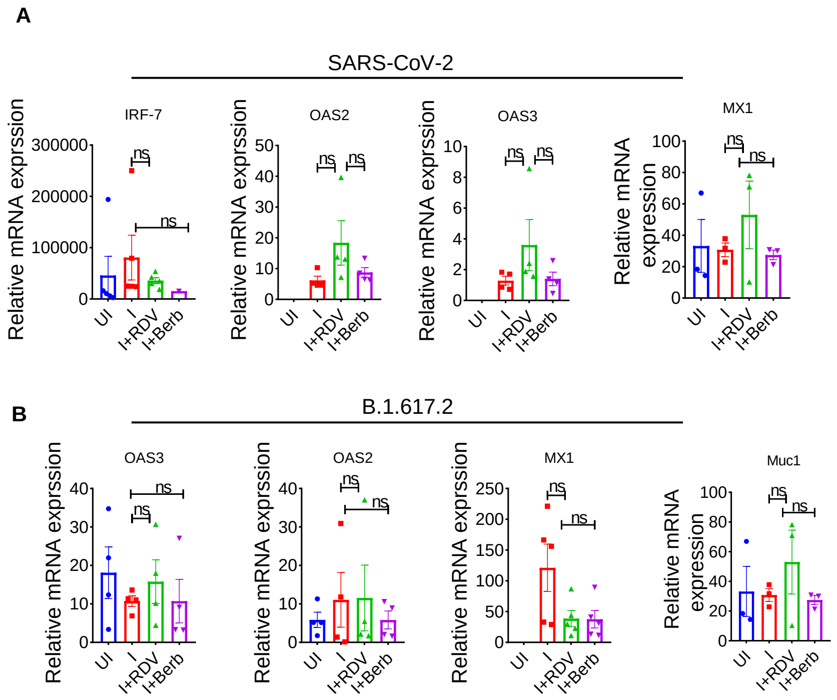

Fig. S5. Antiviral gene expression analysis in vivo, related to Figure 5. A-B) relative gene expression analysis of IRF-7, OAS2, OAS3, MX1 and MUC1 (n=4 mice per group); Data represents ± SEM., One-way ANOVA (Tukey's multiple comparison test). All experiments were performed twice. Statistical analyses are compared with the vehicle-treated group. ns = p > 0.05.
